# Rates, predictors, and mortality of sepsis-associated acute kidney injury: a systematic review and meta-analysis

**DOI:** 10.1186/s12882-020-01974-8

**Published:** 2020-07-31

**Authors:** Jiefeng Liu, Hebin Xie, Ziwei Ye, Fen Li, Lesan Wang

**Affiliations:** 1 Department of epidemiology and health statistics, Xiangya School of public health, Central South University, No. 238, Mayuanling Lane, Furong Middle Road, Kaifu District, Changsha City, Hunan Province China; 2 Science and Education Department of Changsha Central Hospital, Changsha, Hunan China; 3 Shanghai Health Development Research Center, Shanghai City, China

**Keywords:** Acute kidney injury, Sepsis, Risk factor, Meta-analysis, Systematic review

## Abstract

**Background:**

Due to the high incidence and mortality of sepsis-associated acute kidney injury, a significant number of studies have explored the causes of sepsis-associated acute kidney injury (AKI). However, the opinions on relevant predictive risk factors remain inconclusive. This study aimed to provide a systematic review and meta-analysis to determine the predisposing factors for sepsis-associated AKI.

**Method:**

A systematic literature search was performed in the Medline, Embase, Cochrane Library, PubMed, and Web of Science, databases, with an end-date of 25th May 2019. Valid data were retrieved in compliance with specific inclusion and exclusion criteria.

**Result:**

Forty-seven observational studies were included for analysis, achieving a cumulative patient number of 55,911. The highest incidence of AKI was caused by septic shock. Thirty-one potential risk factors were included in the meta-analysis. Analysis showed that 20 factors were statistically significant. The odds ratio (OR) and 95% confidence interval (CI), as well as the prevalence of the most frequently-seen predisposing factors for sepsis-associated AKI, were as follows: septic shock [2.88 (2.36–3.52), 60.47%], hypertension [1.43 (1.20–1.70), 38.39%], diabetes mellitus [1.59 (1.47–1.71), 27.57%], abdominal infection [1.44 (1.32–1.58), 30.87%], the administration of vasopressors [2.95 (1.67–5.22), 64.61%], the administration of vasoactive drugs [3.85 (1.89–7.87), 63.22%], mechanical ventilation [1.64 (1.24–2.16), 68.00%], positive results from blood culture [1.60 (1.35–1.89), 41.19%], and a history of smoking [1.60 (1.09–2.36), 43.09%]. Other risk factors included cardiovascular diseases, coronary artery diseases, liver diseases, unknown infections, the administration of diuretics and ACEI/ARB, the infection caused by gram-negative bacteria, and organ transplantation.

**Conclusion:**

Risk factors of S-AKI arise from a wide range of sources, making it difficult to predict and prevent this condition. Comorbidities, and certain drugs, are the main risk factors for S-AKI. Our review can provide guidance on the application of interventions to reduce the risks associated with sepsis-associated acute kidney injury and can also be used to tailor patient-specific treatment plans and management strategies in clinical practice.

## Background

Sepsis-associated acute kidney injury (S-AKI) is a major public health condition that is associated with a significant disease burden. S-AKI is a syndrome of acute functional impairment and organ damage that could be associated with long-term adverse outcomes. Sepsis is the most common cause of acute kidney injury (AKI) in critically ill patients, and is observed in 40–50% of patients with AKI [1–4]. Of particular importance is the fact that S-AKI is closely associated with poor clinical outcomes. For instance, the mortality rate of sepsis patients with AKI complications is significantly higher than that of non-AKI patients [5]. Among critically ill patients with AKI, S-AKI is correlated with a higher risk of in-hospital death and longer durations in hospital than AKI caused by any other reasons [3]. Despite significant advances in medicine and surgical treatment, the morbidity associated with this condition remains rather high. Mounting evidence suggests that the incidence of AKI incidence is steadily increasing. A previous 10-year cohort study, including more than 90,000 patients from more than 20 ICUs, indicated that the incidence of AKI incidence has increased by 2.8% per year [1]. Moreover, along with the global trend for aging, the majority of patients with sepsis are elderly; furthermore, the number of patients with sepsis-associated AKI is likely to continue to increase [6, 7]. Sepsis-associated AKI is associated with a high burden of morbidity and mortality in both children and adults with critical illness. Unfortunately, the pathogenesis of S-AKI is still not completely understood. There are also difficulties in the early diagnosis and treatment of S-AKI that need to be solved. Therefore, it is vital that we develop tools to identify the risk factors of S-AKI early so that we can attempt to prevent this disease. Although a number of studies have explored the risk factors associated with the development of AKI in patients with sepsis, clinical opinions remain inconclusive due to regional differences and inconsistencies in the diagnostic criteria relating to sepsis and AKI. In this study, we aimed to systematically review previous observational studies (cohort/case-control studies) and to perform meta-analyses with the eligible evidence to investigate the association between sepsis and AKI.

## Methods

### Inclusion criteria

Studies that met the following criteria were included for data extraction: (1) Patients needed to be older than 16 years with a hospitalization stay of greater than 24 h; (2) Studies needed to contain information presented in a 2 × 2 contingency table; (3) Sepsis and septic shock needed to be diagnosed using internationally-recognized standards, such as sepsis 1.0 [8], sepsis 2.0 [9], or sepsis 3.0 [10]; (4) Acute kidney injury needed to be diagnosed using internationally-recognized standards, such as KDIGO, AKIN, and RIFLE; (5) Cohort or case-control studies needed the patients to be grouped into sepsis with AKI and sepsis without AKI.

### Data sources and search strategy

A systematic review and meta-analysis of scientific peer-reviewed literature was performed by following the recommendations from the Preferred Reporting Items for Systematic Reviews and Meta-analysis (PRISMA) guideline (see Additional file 1) [11].

The systematic literature search was performed in Medline, Embase, Cochrane Library, PubMed, and Web of Science, databases from inception to June 2019 with no restrictions. The search aimed to retrieve studies that assessed the risk of AKI development in patients with sepsis. The following search terms were used: (septic OR sepsis OR severe sepsis OR Septicemia OR septic shock OR sepsis-associated OR sepsis-associated) AND (Acute Kidney Injury OR Acute Renal Injury OR Acute Renal Insufficiency OR AKI OR acute renal failure OR ARF). The reference lists of the included articles were also manually retrieved. We did not include gray literature (literature that has not published) or conference abstracts.

### Data extraction

Two independent reviewers participated in the entire process of literature retrieval. First-round screening was performed based on the title and abstract so that we could exclude studies on irrelevant topics. Next, the included articles were screened based on full text; non-eligible articles that did not meet the inclusion criteria were excluded. Data extraction was performed using a standardized data collection form, including: (1) study characteristics: publication year, study design, country of origin, diagnostic criteria for sepsis and acute kidney injury, type of sepsis, period of data report; (2) the number of 2 × 2 contingency tables and unadjusted crude odds ratios with regards to demographic data (gender) and the independent variables/predictors under investigation (comorbidities, source of infection, medication, invasive treatment, types of sepsis, and blood culture); and (3) outcome: the primary endpoint was S-AKI; the secondary outcome was the prevalence of influential factors and mortality in patients with S-AKI.

### Quality assessment

Study selection, data extraction, and quality assessments were independently performed by two authors. Any disagreements were resolved through discussions until a consensus was reached. If disagreements persisted, another reviewer would be invited to the discussion to achieve a final consensus. Quality assessment of the observational studies that were included in the meta-analysis was performed using the Newcastle-Ottawa scale (available at http://www.ohri.ca/programs/clinical_epidemiology/oxf ord.asp).

### Statistical analysis

The core characteristics of the study and patients were sorted and summarized. The frequency distribution was expressed as a percentage. For meta-analysis, we only used non-adjusted crude odds ratios (OR) from no less than 3 studies to standardize the results; this was due to the significant variability of multivariable models across different studies. Stata/SE version 11 was used for all statistical analyses and a two-sided *P* value of 0.05 or less was considered to be statistically significant. Heterogeneity among studies was evaluated by calculating the *I*^2^ statistic (significance level was set to I^2^ > 50%) and chi-squared value (significance level set to *P* < 0.10). I^2^ values of 25 and 75% were used as the criteria for classifying the degree of inter-trial heterogeneity (I^2^ < 25%: low heterogeneity; I^2^ > 25% and < 75%: moderate heterogeneity: I^2^ > 75%: high heterogeneity). If severe heterogeneity was present at I^2^ > 50%, then a random-effects model was selected, otherwise the fixed-effects model was used. For results with a heterogeneity of < 50% and a fixed-effects model, the stability would be explored by transformation into a random-effects model. Meta regression and subgroup analyses (≥ 6 studies) were conducted according to publication year, study design, country of origin, sepsis type, and the diagnostic criteria used for acute kidney injury and sepsis, on the condition of high inter-trial heterogeneity (I^2^ > 50% and P < 0.10). Sensitivity analysis of the overall risk (≥ 3 studies) was conducted by omitting 1 study each time in order to estimate the impact of individual studies. Publication bias was examined visually by the use of funnel plots, and the Egger’s test was used to carry out asymmetric tests on the pooled data of ≥7 studies.

## Results

### Literature search (Fig. 1)

In total, 8033 records were initially identified from the Medline, Embase, Cochrane Library, PubMed, and Web of Science, databases. By filtering the title and abstract, we were able to exclude duplicate articles, review studies, and studies on unrelated topics. In total, the full text of 626 studies was reviewed. After excluding comment papers, studies with inconsistent control settings, articles with unspecified AKI or sepsis diagnostic criteria, studies performed in special populations, and those with limited data, 47 articles met the inclusion criteria and were included in the systematic review and meta-analysis.

**Fig. 1:**
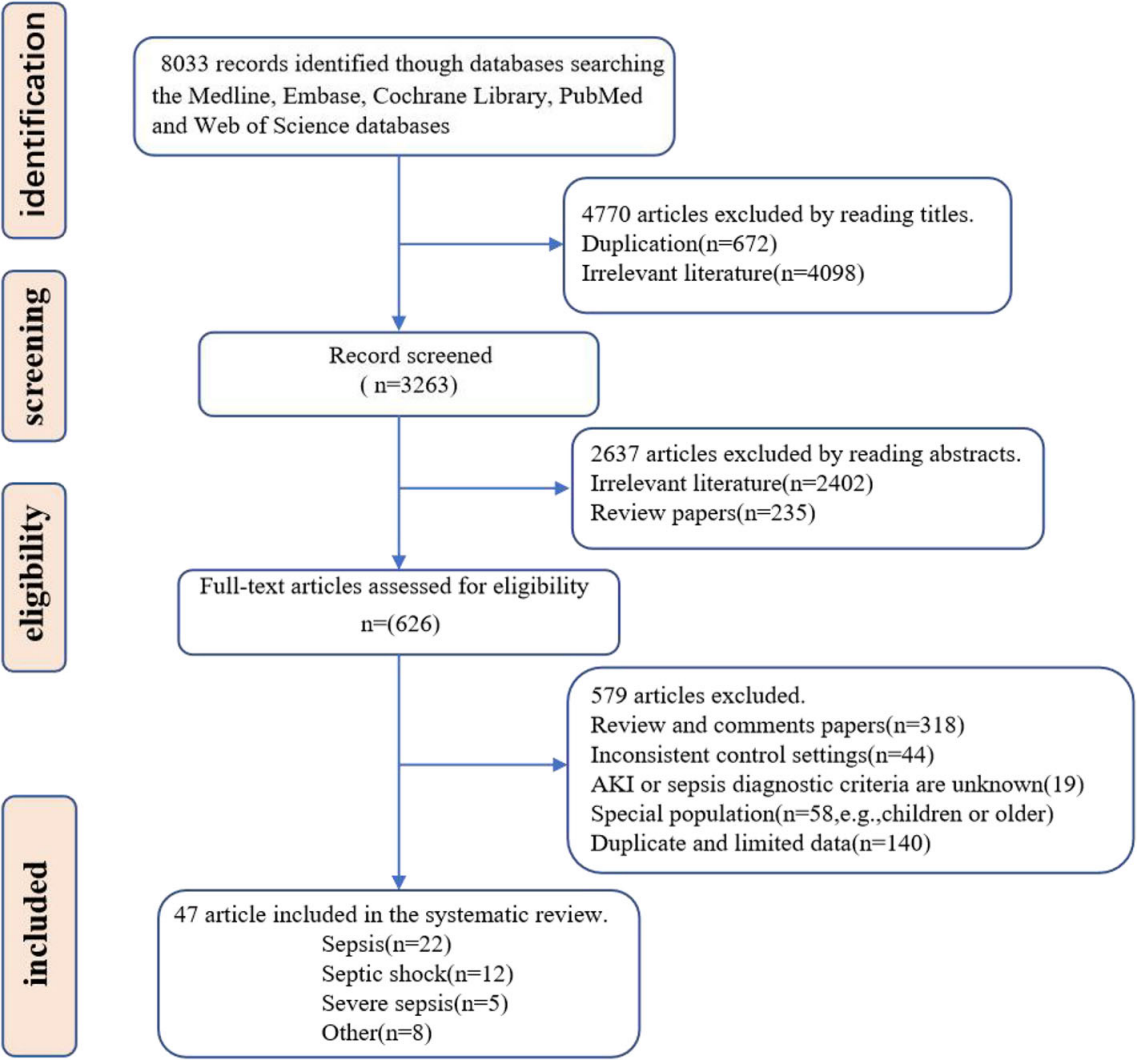
Flow diagram showing search strategy and study selection. The diagram shows the numbers of titles and studies reviewed in preparation of this meta-analysis of development of acute kidney injury in sepsis patients. n represents the number of studies included in data syntheses. Eight included ‘other’ article represent that the study subjects included at least two of three sepsis (sepsis, septic shock and severe sepsis)

### Characteristics of the included studies (Table 1)

The characteristics of the included articles are shown in Table 1. Studies were published between 2008 and 2019 and originated from 18 countries (Spain, Greece, United Kingdom, France, Netherlands, Sweden, Canada, United States, Brazil, China, Japan, Saudi Arabia, Turkey, Finland, Portugal, South Korea and Australia) on four continents (Europe, America, Asia and Oceania). Overall, we included 12 retrospective cohort studies, 25 prospective cohort studies, and 12 case-control studies, with a total of 55,911 patients with sepsis. Document quality assessment showed that the methodological quality of all of the included studies was high, achieving a quality score of 8 (≥ 6).

**Table 1:** Characteristics of included studies in systematic review and meta-analysis

Author	Plublication year	Country	AKI diagnostic criteria	Sepsis types	Study period	Research design	Numble AKI VS No AKI	Quality score
Bu et al. [12]	2019	China	KDIGO	Sepsis and Septic shock	2015–2017	Retrospective case-control study	132/90	7
Hsu et al. [13]	2019	China	AKIN	Sepsis	2012–2016	Retrospective case-control study	99/597	6
Vilander et al. [14]	2019	Finland	KDIGO	Sepsis	2011–2012	Prospective cohort study	300/353	7
Xing et al. [15]	2019	China	KDIGO	Septic shock	2018.8–2018.11	Prospective cohort study	29/43	8
Moman et al. [16]	2018	USA	KDIGO	Septic shock	2007–2009	Retrospective cohort study	160/73	8
Zhi et al. [17]	2018	China	AKIN	Sepsis	2009–2015	Retrospective case-control study	315/267	5
Zhou et al. [18]	2018	China	AKIN	Sepsis	2010–2017	Retrospective case-control study	405/348	6
Costa et al. [19]	2018	Brazil	KDIGO	Septic shock	2014–2015	Prospective cohort study	66/63	7
Song et al. [20]	2018	China	KDIGO	Sepsis	2015–2016	Prospective cohort study	52/72	7
Hu et al. [21]	2018	China	RIFLE	Sepsis	2016–2017	Prospective cohort study	52/53	8
Fatani et al. [22]	2018	Saudi Arabia	RIFLE	Severe sepsis and Septic shock	2016–2017	Prospective cohort study	127/73	7
Gameiro et al. [23]	2017	Portugal	KDIGO	Sepsis and Septic shock	2008–2014	Retrospective case-control study	399/57	6
Katayama et al. [24]	2017	Japan	KDIGO	Sepsis	2011–2016	Retrospective case-control study	163/351	7
Vilander et al. [25]	2017	Finland	KDIGO	Septic shock	2011–2012	Prospective cohort study	252/226	7
Suberviola et al. [26]	2017	spain	KDIGO	Septic shock	2005–2010	Prospective cohort study	312/74	7
Fisher et al. [27]	2017	Sweden	KDIGO	Septic shock	–	Prospective cohort study	225/71	6
Pérez-Fernández et al. [28]	2017	USA	KDIGO	Severe sepsis and Septic shock	2005–2007	Prospective cohort study	82/178	7
Pereira et al. [29]	2017	Portugal	REFILE	Severe sepsis and Septic shock	2008–2014	Retrospective case-control study	384/72	7
Panich et al. [30]	2017	Thailand	AKIN	Sepsis	2014–2014	Prospective cohort study	79/60	7
Su et al. [31]	2016	China	KDIGO	Severe sepsis	–	Prospective cohort study	45/27	6
Yilmaz et al. [32]	2015	Turkey	AKIN	Severe sepsis	2011–2013	Retrospective cohort study	68/50	7
Medeiros et al. [33]	2015	Japanese	AKIN	Sepsis	2013–2014	Retrospective cohort study	144/56	8
Dai et al. [34]	2015	China	KDIGO	Sepsis	2012–2014	Prospective cohort study	55/57	7
Sood et al. [35]	2014	Canada	RIFLE	Septic shock	1996–2008	Prospective cohort study	3298/1195	7
Peng et al. [36]	2014	China	KDIGO	Sepsis	2008–2011	Prospective cohort study	101/110	8
Patschan et al. [37]	2014	Germany	AKIN	Sepsis	–	Retrospective case-control study	22/11	7
Tu et al. [38]	2014	China	AKIN	Sepsis	2011–2013	Prospective cohort study	49/101	6
Fan et al. [39]	2014	China	RIFLE	Sepsis	2012–2014	Prospective cohort study	58/67	7
CHO et al. [40]	2014	Korea	RIFLE	Sepsis	2010–2011	Prospective cohort study	44/18	7
Terzi et al. [41]	2014	Greece	RIFLE	Sepsis	–	Prospective cohort study	16/29	6
Poukkanen et al. [42]	2013	Finland	KDIGO	Severe sepsis	2011–2012	Retrospective case-control study	153/270	7
Legrand et al. [43]	2013	France	AKIN	Severe sepsis and Septic shock	2006–2010	Prospective cohort study	69/68	8
Cardinal-Fernández et al. [44]	2013	Spain	RIFLE	Severe sepsis	2005–2008	Prospective cohort study	65/74	7
de Geus et al. [45]	2013	Netherlands	AKIN	Sepsis	2007–2008	Prospective cohort study	49/432	7
Katagiri et al. [46]	2013	Japan	RIFLE	Sepsis	2010–2011	Prospective cohort study	24/10	6
Aydogdu et al. [47]	2013	Turkey	RIFLE	Sepsis	2008–2010	Prospective cohort study	63/66	7
Suh et al. [48]	2013	South Korean	RIFLE	Sepsis and Septic shock	2010	Retrospective case-control study	573/419	8
Poukkanen et al. [49]	2013	Finland	KDIGO	Severe sepsis	2011–2012	Retrospective case-control study	437/393	7
Zhao et al. [50]	2013	China	AKIN	Sepsis	2011–2013	Retrospective case-control study	90/58	6
Payen et al. [51]	2012	Brazil	AKIN	Severe sepsis and Septic shock	2004–2005	Retrospective cohort study	129/47	6
Frank et al. [52]	2012	USA	AKIN	Septic shock	1999–2009	Retrospective cohort study	627/637	7
Plataki et al. [53]	2011	USA	RIFLE	Septic shock	2005–2007	Retrospective cohort study	237/153	7
Ma°rtensson et al. [54]	2010	Sweden	RIFLE OR AKIN	Septic shock		Prospective cohort study	18/7	6
YANG et al. [55]	2009	China	AKIN	Septic shock	2001–2008	Retrospective cohort study	126/32	7
Lopes et al. [56]	2009	Portugal	AKIN	Sepsis	2004–2007	Retrospective cohort study	99/216	7
Bagshaw et al. [57]	2009	Canada, the United States and SaudiArabia	RIFLE	Septic shock	1989–2005	Retrospective cohort study	2917/1615	7
Bagshaw et al. [58]	2008	Australia	RIFLE	Sepsis	2000–2005	Retrospective cohort study	14,039/19336	8

### Summary data from the included studies (Table 2)

This study summarized the characteristics of sepsis patients who developed AKI. ICU mortality, hospital mortality, 28-day mortality, and 90-day mortality, of patients with S-AKI were respectively reported at 45.99% (1989/4325 cases) in 15 studies, 49.84% (2732/5481) in 10 studies, 36.67% (161/439) in 4 studies, and 64.66% (2406/3721) in 5 studies., respectively. In S-AKI patients, the highest mortality rate for AKI was caused by septic shock; severe sepsis was associated with the lowest mortality rate.

**Table 2:** Summary data of all sepsis patients who developed AKI from included studies

Characteristic	No.Studies	Prevalence	Sepsis		Septic shock		Severe sepsis	
			No.Studies	Prevalence	No.Studies	Prevalence	No.Studies	Prevalence
Septic AKI	47	48.73% (27,248/55911)	22	41.98% (16,399/39067)	12	60.47%(12,678/20965)	5	38.92% (768/1570)
Sex (male)	44	59.70% (5913/9904)	22	63.68% (1380/2167)	11	59.64% (3191/5350)	5	64.45% (495/768)
Comorbidities								
Hypertension	32	38.39% (3263/8500)	14	42.28% (859/1817)	6	26.16% (1073/4102)	5	58.07% (446/768)
Diabetes mellitus	32	27.57% (2248/8155)	13	20.53% (373/1817)	7	26.75% (1897/7091)	5	30.20% (232/768)
Stroke	4	22.79% (67/294)	1	22.33% (67/300)	–	–	1	17.78% (8/45)
Cancer	6	18.23% (705/3745)	–	–	2	18.80% (650/3458)	1	16.33% (8/49)
Chronic kidney disease	14	16.46% (449/2795)	7	15.52% (178/1147)	2	45.13% (102/226)	2	11.02% (65/590)
Cardiovascula disease	11	16.30% (2522/15477)	4	19.47% (169/868)	–	–	1	7.00% (3/45)
Congestive heart failure	7	12.69% (491/3869)	2	17.26% (39/226)	4	12.64% (446/3529)	1	8.80% (6/68)
COPD	17	12.41% (1114/8976)	6	12.69% (90/709)	5	12.99% (873/6721)	1	5.20% (25/437)
Hepatic failure	4	12.16% (449/3691)	2	39.76% (134/337)	1	9.90% (290/2917)	3	12.61% (83/658)
Coronary artery disease	8	11.58% (457/3948)	4	10.14% (88/868)	2	9.30% (274/2946)	1	6.15% (4/65)
Systolic heart failure	4	11.25% (135/1200)	1	8.00% (24/300)	2	14.32% (59/412)	1	11.90% (52/437)
Immnosuppression	7	10.35% (1888/18249)	2	12.74% (1300/14204)	3	15.80% (550/3481)	1	7.20% (35/437)
Cirrhosis	6	4.71% (99/2104)	1	1.73% (7/405)	2	7.50% (59/787)	–	–
Liver disease	7	3.74% (554/14081)	3	3.57% (509/14282)	1	8.73% (22/252)	2	8.59% (17/198)
Admission category								
Emergency admission	7	50.88% (9235/18149)	2	50.90% (7298/14339)	2	41.46% (1314/3169)	2	97.12% (573/590)
Medical admission	8	47.02% (8701/18506)	3	49.16% (6938/14112)	2	36.99% (1311/3544)	–	–
Operative admission	5	30.91% (353/1142)	1	22.33% (67/300)	1	23.02% (58/252)	2	28.81% (170/590)
Surgical ward	7	17.73% (3787/21359)	3	16.51% (2375/14388)	3	21.29% (1380/6482)	–	–
Source of infection								
Pulmonary	19	46.05% (1480/3214)	8	57.96% (448/773)	5	41.10% (603/1467)	3	48.02% (316/658)
Respiratory	7	32.08% (273/85)	2	41.22% (54/131)	2	32.74% (74/226)	2	26.36% (29/110)
Abdominal	25	30.87% (2152/6971)	7	32.12% (177/551)	7	28.16% (1253/4450)	5	28.65% (220/768)
Urinary tract	19	11.14% (630/5653)	6	12.01% (58/483)	6	11.34% (483/4259)	5	11.38% (80/703)
Skin or soft tissue	13	6.03% (335/5554)	3	2.15% (5/232)	4	5.40% (218/4033)	3	10.71% (68/635)
Unknow	4	6.02% (100/1662)	–	–	2	8.30% (73/879)	–	–
Community acquired	3	57.36% (2041/3558)	–	–	1	56.80% (1657/2917)	2	65.08% (384/590)
Nosocomial acquired	2	39.81% (2474/6215)	–	–	2	39.81% (2474/6215)	–	–
Medications								
Vasopressors	7	64.61% (1293/2001)	3	45.04% (100/222)	2	59.38% (513/864)	–	–
vasoactive drugs	5	63.22% (911/1441)	2	35.69% (131/367)	1	67.50% (108/160)	2	96.44% (569/590)
Steroids	3	30.80% (85/276)	2	38.16% (79/207)	–	–	–	–
Diuretics	4	30.77% (296/962)	–	–	1	39.40% (97/252)	2	30.85% (182/590)
ACEI or ARB	8	25.62% (619/2416)	1	18.41% (58/315)	3	24.97% (200/801)	3	33.59% (220/655)
Stains	5	21.77% (357/1640)	–	–	2	24.13% (118/489)	1	15.79% (69/437)
Nsaids		9.63% (203/2108)	1	16.19% (51/315)	2	11.45% (56/489)	2	12.54% (74/590)
Bacteria								
Gram-negative bacteria	3	17.26% (160/927)	–	–	1	22.3% (49/225)	–	–
Gram-positive bacteria	4	10.43% (99/949)	1	18.20% (4/22)	1	28.6% (63/225)	–	–
Invasive treatment								
Mechanical ventilation	23	68.00% (7167/10539)	7	49.17% (415/844)	6	71.21% (5481/7643)	4	75.25% (529/703)
renal replacement therapy	6	39.51% (320/810)	1	36.53% (19/52)	1	18.18% (12/66)	–	–
Dialysis	3	28.92% (59/204)	2	35.04% (48/137)	–	–	–	–
Blood transfusion	3	19.46% (94/483)	1	7.64% (11/144)	2	27.39% (3/303)	–	–
Organ transplant	3	3.76% (252/6703)	–	–	2	3.94% (245/6215)	1	1.60% (7/437)
Positive blood culture	8	41.38% (3259/7876)	–	–	4	42.89% (2836/6612)	2	30.29% (146/482)
Bloodstream infection	4	6.61% (237/3586)	1	17.31% (9/52)	1	7.40% (216/2917)	1	4.70% (6/437)
Smoke history	5	43.09% (642/1490)	2	40.42% (291/720)	–	–	1	32.35% (22/68)
ARDS	3	47.02% (489/1040)	1	81.19% (82/101)	2	43.34% (407/939)	–	–
Multiorgan dysfunction (≥3)	3	50.11% (436/870)	1	70.48% (222/315)	–	–	–	–
Mortality								
ICU mortality	10	45.99% (1989/4325)	2	50.00% (46/92)	4	50.47% (1672/3313)	1	35.38% (23/65)
Hospital mortality	15	49.84% (2732/5481)	7	42.17% (245/581)	3	55.83% (1935/3466)	1	29.29% (128/437)
28-day mortality	4	36.67% (161/439)	1	30.61% (15/49)	1	71.42% (90/126)	–	–
90-day morality	5	64.66% (2406/3721)	–	–	1	58.42% (1704/2917)	2	40.0% (236/590)

*COPD* chronic obstructive pulmonary disease, *ACEI or ARB* angiotensin converting enzyme inhibitors or Angiotensin Receptor Blocker, *ARDS* acute respiratory distress syndrome

The most prevalent comorbidity was ARDS (47.02%; 489/1040; from 3 studies), followed by hypertension (38.39%; 3263/8500; from 32 studies), diabetes (27.57%; 2248/8155; from 32 studies) and stroke (22.79%; 67/294;from 4 studies), while cirrhosis and liver disease accounted for only 4.71% (99/2104; from 6 studies) and 3.74% (554/14081; from 7 studies), respectively. Hepatic failure was more common in patients with sepsis compared with those with septic shock and severe sepsis. Hypertension in patients with septic shock was less common than sepsis and severe sepsis (26.16% vs 42.28 and 58.07%), while chronic kidney disease was more prevalent (45.13% vs 15.52 and 11.02%). Hypertension and diabetes were more prevalent in patients with severe sepsis than in sepsis and septic shock (58.7% vs 42.28 and 26.16%, 30.20% vs 20.53 and 26.75%).

On admission, patient source mainly included emergency admission (50.88%; 9235/18149; from 8 studies) and medical admission (47.02%; 8701/18506; from 7 studies), followed by operative admission and surgical ward. Vasoactive drugs were the most commonly used drugs, accounting for 64.61% of cases (1293/2001; from 5 studies), of which vasopressors were the most frequently used, accounting for 63.22% of cases (911/1441; from 7 studies), followed by steroids, diuretics, ACEI or ARB, stains, and NSAIDS. Vasoactive drugs and vasopressors were more prevalent in patients with septic shock and severe sepsis than in patients with sepsis.

Six sources of infection were reported in this study, including pulmonary infection (46.05%; 1480/3214; from 19 studies), respiratory infection (32.08%; 85/273; from 7 studies), abdominal infection (30.87%; 2152/6971; from 25 studies), urinary tract infection (11.14%; 630/5653; from 19 studies), skin or soft tissue infection (6.03%; 335/5554; from 13 studies), and unknown infections (6.02%; 100/1662; from 4 studies).

Community acquired infection was reported in 3 studies with a prevalence of 57.36% (2041/3558), which was higher than nosocomial acquired infection, reported in 2 studies (39.81%; 2474/6215). Twenty-four studies reported mechanical ventilation in 68.00% of patients (7167/10539; from 24 studies), and mechanical ventilation was more frequently used in patients with septic shock and severe sepsis compared with patients with sepsis. Other prevalent factors included positive blood culture (41.38%; 3259/7876; from 8 studies) and smoking history (43.09%; 642/1490; from 5 studies).

### Risk factors for AKI (Fig. 2)

#### Comorbidities

Pooled data from 32 studies indicated that hypertension was a significant predictor (OR: 1.43; 95% CI: 1.20–1.70) with moderate heterogeneity (I^2^ = 74.00%). The source of heterogeneity was not identified by subgroup analysis. The results of the sensitivity analyses were consistent. After excluding 3 studies with rather high heterogeneity, the level of heterogeneity decreased, and the result remained stable (see Additional file 2).

**Fig. 2:**
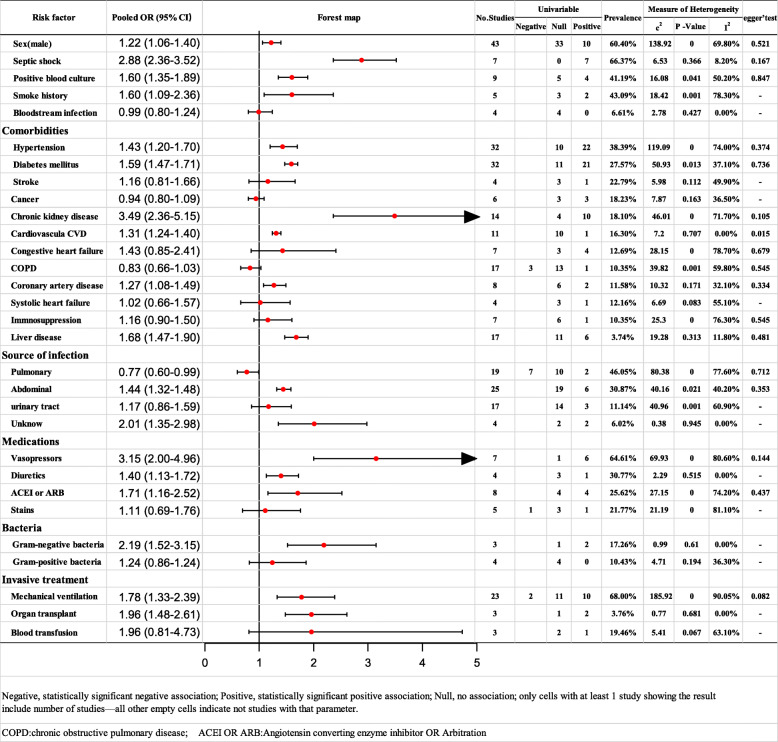
Meta-analysis of risk factors of AKI

Pooled data from 32 studies indicated that diabetes mellitus was a significant predictor (OR: 1.59; 95% CI: 1.47–1.71) with moderate heterogeneity (I^2^ = 37.1%). The results remained stable even with a random-effects model (see Additional file 3).

Pooled data from 14 studies indicated that chronic kidney disease was a significant predictor (OR: 3.49; 95% CI: 2.36–5.15) with moderate heterogeneity (I^2^ = 71.70%). The source of heterogeneity was not identified by subgroup analysis. The results of sensitivity analyses were consistent. After excluding one study with high heterogeneity, I^2^ was reduced to 25.6% (low heterogeneity) and the result remained stable (see Additional file 4).

Cardiovascular disease (from 14 studies; OR: 1.31; 95% CI: 1.24–1.40) and liver disease (from 17 studies; OR: 1.68; 95% CI: 1.47–1.90) were identified as risk factors with low levels of heterogeneity. The results remained stable even with a random-effects model (see Additional files 5 and 6).

Pooled data from 8 studies indicated that coronary artery disease was a significant predictor (OR: 1.27; 95% CI: 1.08–1.49) with moderate heterogeneity (I^2^ = 37.1%). The results remained stable with the random-effects model (see Additional file 7).

#### Source of infection

Pooled data from 8 studies indicated that pulmonary infection was a significant predictor (OR: 0.77; 95% CI: 0.60–0.99) with moderate heterogeneity (I^2^ = 77.60%). The source of heterogeneity was not identified by subgroup analysis. The results of sensitivity analyses were consistent (see Additional file 8).

Pooled data from 25 studies indicated that abdominal infection was a significant predictor (OR: 1.44; 95% CI: 1.32–1.58) with moderate heterogeneity (I^2^ = 40.20%). The results of the sensitivity analyses were consistent. After excluding one study with high levels of heterogeneity, the results remained stable; the results were also stable with the fixed-effects model (see Additional file 9).

Pooled data from 25 studies indicated that unknown infection was a significant predictor (OR: 2.01; 95% CI: 1.35–2.98) with low heterogeneity (I^2^ = 0%). The results were still stable with the random-effects model (see Additional file 10).

#### Medications

Vasopressors (from 7 studies; OR: 3.15; 95% CI: 2.00–4.96) and ACEI or ARB (from 8 studies; OR: 1.61; 95% CI: 1.10–2.36) were all identified as risk factors with high levels of heterogeneity (I^2^ ≥ 75%). The source of heterogeneity was not identified by subgroup analysis and the sensitivity analyses were stable (see Additional file 11).

**Fig. 3:**
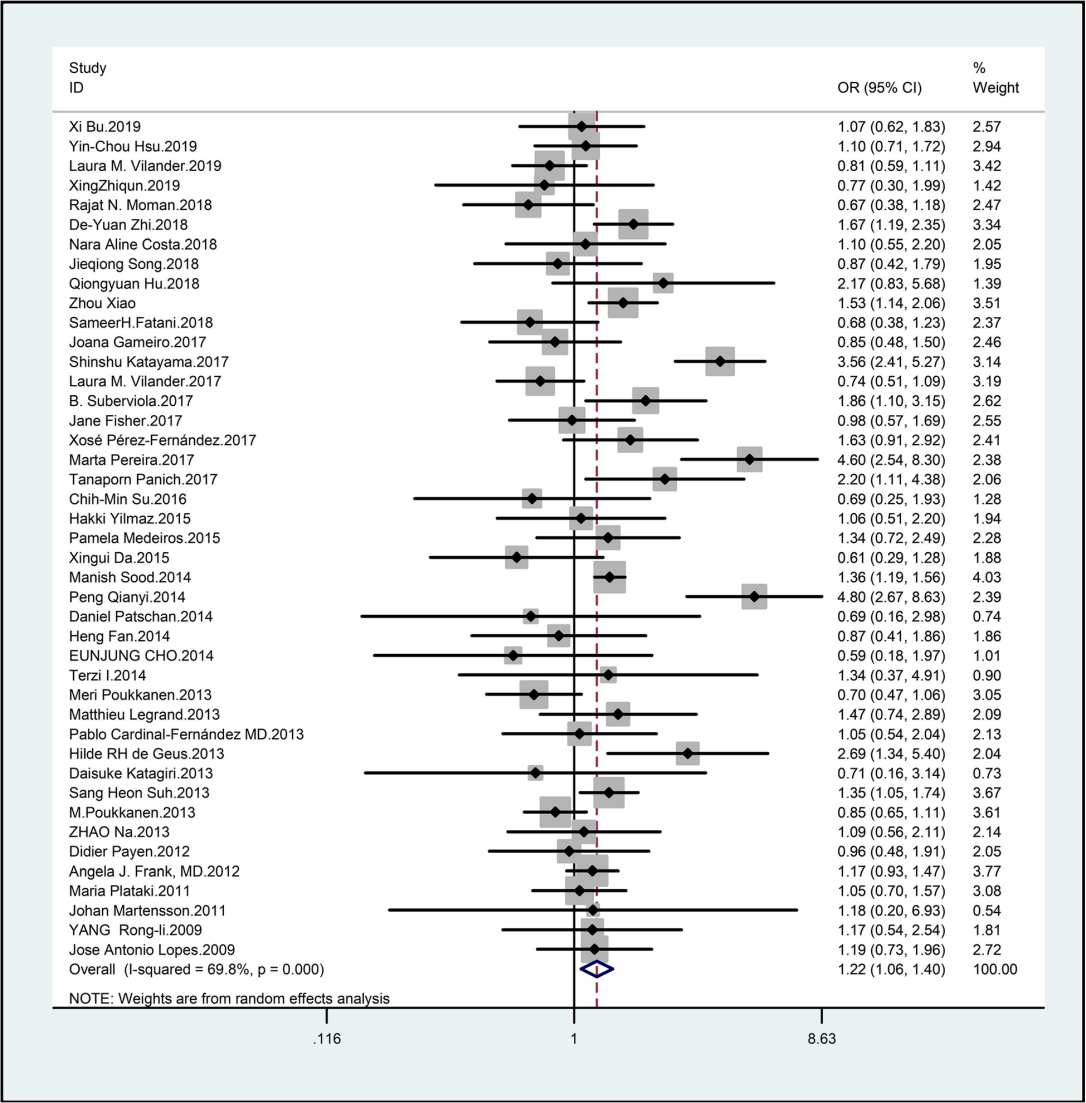
Forest plot for meta-analysis of the association of male sex and AKI

Pooled data from 5 studies indicated that diuretics were a significant predictor (OR: 1.40; 95% CI: 1.13–1.72) with low levels of heterogeneity (I^2^ = 0%). The results remained stable with the random-effects model (see Additional file 12) (Fig. 3).

#### Other factors

Pooled data from 43 studies indicated that male gender was a significant predictor (OR: 1.22; 95% CI: 1.06–1.40) with moderate heterogeneity (I^2^ = 69.80%). The source of heterogeneity was not identified by subgroup analysis. The results arising from sensitivity analyses were consistent (see Additional file 13).

Pooled data from 9 studies indicated that positive blood culture was a significant predictor (OR: 1.60; 95% CI: 1.35–1.89) with moderate heterogeneity (I^2^ = 50.20%). The source of heterogeneity was not identified by subgroup analysis. The sensitivity analysis results were consistent (see Additional file 14).

Pooled data from 5 studies indicated that smoking history was a significant predictor (OR: 1.60; 95% CI: 1.09–2.36) with high heterogeneity (I^2^ = 78.30%). Results arising from sensitivity analysis were consistent. After excluding one study with high levels of heterogeneity, the result remained stable (see Additional file 15).

Pooled data from 7 studies indicated that septic shock was a significant predictor (OR: 1.40; 95% CI: 1.13–1.72) with low heterogeneity (I^2^ = 8.2%). The results were still stable with the random-effects model (see Additional file 16).

Gram-negative bacteria (from 3 studies; OR: 2.19; 95% CI: 1.52–3.15) and organ transplantation (from 3 studies; OR: 1.96; 95% CI: 1.48–2.61) were all identified as risk factors with low levels of heterogeneity (I^2^ = 0%); the results remained stable with the random-effects model (see Additional files 17 and 18).

**Fig. 4:**
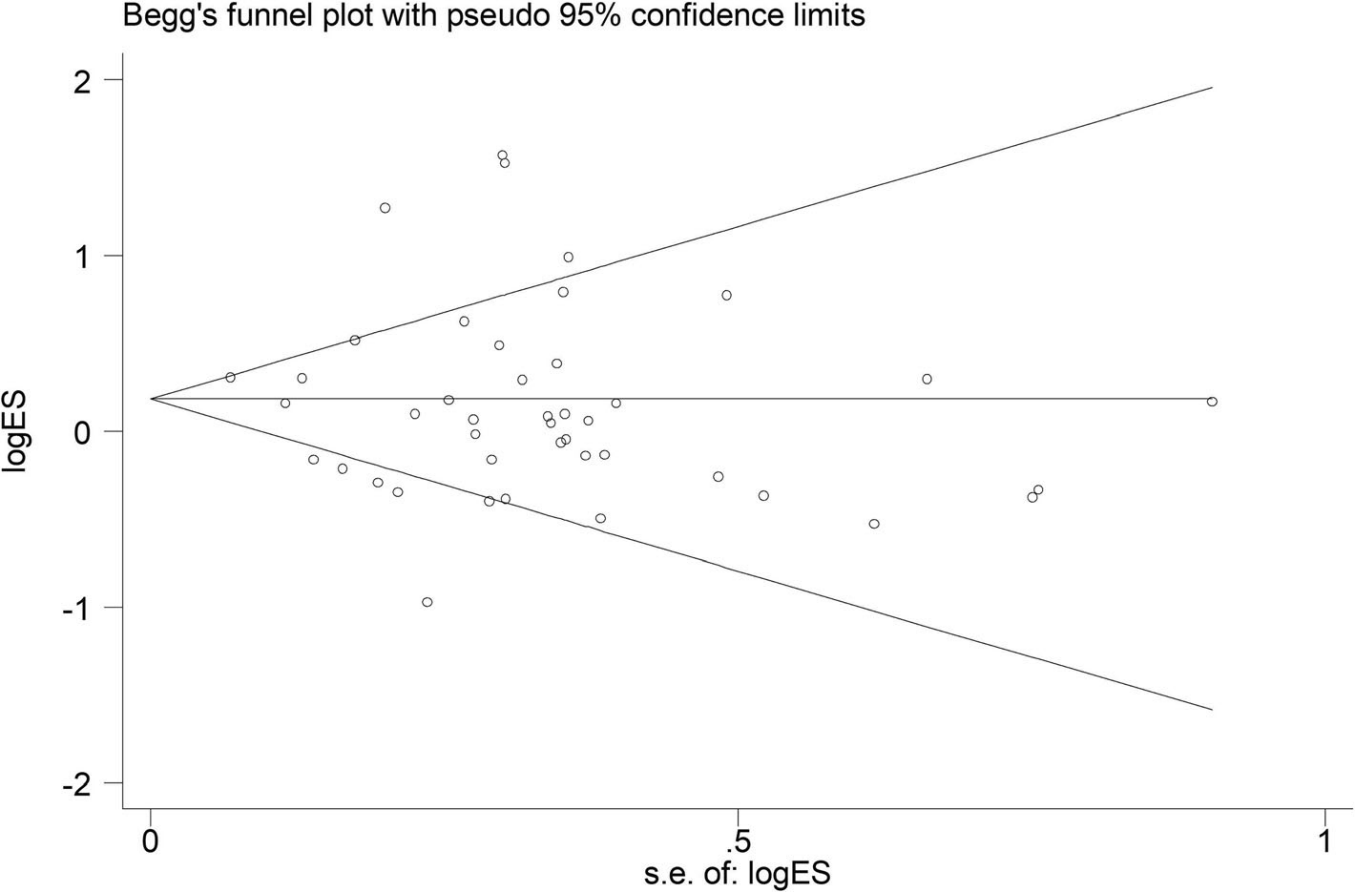
Funnel plot to detect publication bias for male sex, Egger test, *P* = 0.32

Pooled data from 24 studies indicated that mechanical ventilation was a significant predictor (OR: 1.64; 95% CI: 1.24–2.16) with high levels of heterogeneity (I^2^ = 88.70%). The source of heterogeneity was not identified by subgroup analysis. The sensitivity analysis results were consistent (see Additional file 19) (Fig. 4).

### Tests for publication Bias (Fig. 2)

**Fig. 5:**
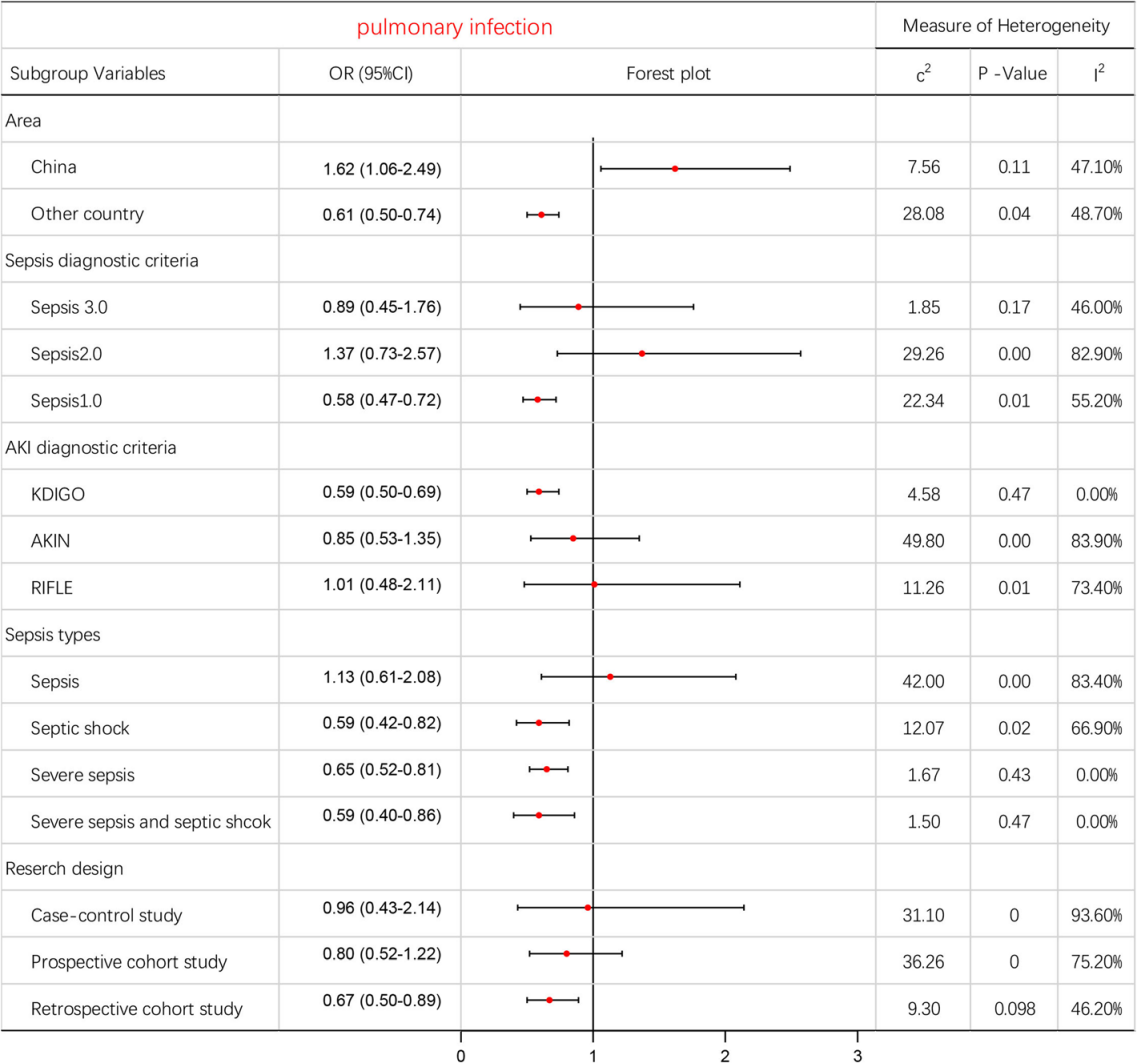
Subgroup analyzes for meta-analysis of the association of pulmonary infection and AKI

Egger’s rank correlation test and Egger’s linear regression test indicated that there was no publication bias for any of the risk factors (≥7 studies) except for cardiovascular disease (*P* = 0.015). Due to the limited number of studies (<7 studies), publication bias was not evaluated for smoking history, cirrhosis, multiorgan dysfunction (≥3), unknown infection, the administration of vasoactive drugs, the use of diuretics, and organ transplantation (Fig. 5).

## Discussion

### Major findings

To the best of our knowledge, this is the first meta-analysis to provide a comprehensive insight into the risk factors associated with AKI in patients with sepsis. In total, 47 studies, including 55,911 patients with sepsis, were included in this systematic review, along with 46 risk factors. The results showed that 19 factors were significant, including comorbidities, sources of infection, medications, and invasive treatments. Risk factors for S-AKI arise from a wide range of sources, making it difficult to predict and prevent this disease. We found that AKI caused by septic shock had the highest incidence and mortality in patients with sepsis. We also found significant inter-trial heterogeneity in studies exploring the association between sepsis and AKI, thus resulting in reduced evidential power. Inevitably, this has led to controversial opinions regarding the risk factors for AKI in patients with sepsis. We, therefore, hope that more homogeneous research can be carried out in the future and more reliable conclusions can be obtained.

### Analysis of risk factors

Risk factors for sepsis-associated AKI can be categorized as pre-sepsis risk factors, sepsis disease-related factors and sepsis-related treatment factors. The pre-sepsis risk factors (e.g., concurrent chronic diseases, gender, age, and smoking history) and sepsis disease itself (e.g., sepsis type, source of infection, and bacterial infection) cannot be altered since they existed at the time of diagnosis. However, these factors can be used to identify patients who are at high risk of AKI, so that timely precautions can be applied accordingly to reduce potential risks in the future. On the other hand, the risk factors associated with sepsis-related treatment can be manually controlled by using efficient strategies (e.g., medication and mechanical ventilation).

#### Pre-sepsis risk factors

Our study showed that multiple chronic comorbidities were associated with the development of AKI in patients with sepsis. Hypertension and diabetes mellitus were the most common risk factors for AKI among all comorbidities; other factors included chronic kidney diseases, cardiovascular diseases, coronary artery diseases, and liver diseases. This may be due to the fact that the majority of patients with sepsis were older adults aged 65 years and older [6, 59]. We found that diabetes mellitus and hypertension were associated with higher risks of AKI; these findings were consistent with those of previous studies [60–63]. Chronic kidney disease has been recognized as a significant risk factor for AKI [64, 65]. Moreover, when AKI occurs in CKD patients, it is more severe and difficult to recover from. There is increasing recognition that AKI and chronic kidney disease (CKD) are closely linked and are likely to promote one another. However, the association between the severity of CKD (e.g., as measured by levels of estimated GFR) and the risk of AKI has not been quantified, although a recent meta-analysis showed that CKD may increase the risk of AKI in patients with diabetes or hypertension. Therefore, in addition to directly increasing the risk of AKI, diabetes mellitus, hypertension, and CKD, could also interact to promote the development of AKI [63]. Furthermore, these three factors are also prevalent risk factors for AKI. Consequently, more attention should be paid to patients with these three risk factors in order to avoid the potential risks of AKI.

Opinions regarding the association between gender and AKI remain controversial, although our study found that male patients may be at a slightly higher risk of AKI compared with their female counterparts. A previous study found that a lower glomerular filtration rate (eGFR) and a higher albumin-creatinine ratio (ACR) were associated with higher AKI risks in both men and women, and that male gender was associated with a higher risk of AKI with a slight attenuation in a lower eGFR but not with a higher ACR [66].

#### Sepsis-disease-related risk factors

Among the patients with sepsis that were included in the present study, we found that AKI caused by septic shock had the highest incidence and mortality, and that septic shock was also a significant risk factor for AKI. Consequently, more attention should be paid to the prevention of AKI in patients with septic shock.

Our data analysis indicated that pulmonary and abdominal infections were the most common source of infection in patients with sepsis who developed AKI. We also found that both of these conditions were associated with the development of AKI. Abdominal infections could increase the risk of AKI development, although our study found that lung infection was a protective factor for AKI, although further research is needed to elucidate the mechanisms underlying this observation. Considering the high levels of heterogeneity (I^2^ = 77.6%), we performed sensitivity analyses an obtained stable results. Subgroup analysis showed different results when considering Chinese populations and other populations. Pulmonary infection was found to be a risk factor in the Chinese population (OR: 1.62; 95% CI: 1.06–2.49) but was a protective factor in other populations (OR: 0.61; 95% CI: 0.50–0.74). We were cautious about the overall results and the results of our subgroup analysis since there was a lack of reasonable interpretation for these results, as well as heterogeneity among different populations. Further research is required to investigate these issues further.

The specific relationship between the occurrence of AKI and bacterial infection has rarely been reported. Our study found that gram-negative bacteria may represent a risk factor for AKI. However, it remains unclear which gram-negative bacteria could be involved. Only one study showed that *Escherichia coli* may be associated with the development of AKI [66]. Further research is now needed to investigate this relationship further.

#### Sepsis-related treatment risk factors

Our study found that diuretics, vasopressors, and ACEI or ARB, could be associated with the occurrence of AKI. Vasoactive drugs are commonly used in patients with sepsis, especially septic shock. Our research found that vasopressors increased the risk of AKI, while the association between AKI and other vasoactive medications remains uncertain. A large cohort study previously showed that ACEI/ARB could be associated with a small increase in the risk of AKI while individual patient characteristics were much more closely correlated with the incidence of AKI [67]. Among patients with CKD, there was no increased risk of developing AKI compared with those who were not exposed to ACEI/ARB, while exposure to ACEI/ARB in patients without CKD increased the risk of AKI. An earlier multi-center prospective study in Shanghai showed that diuretics accounted for 22.2% of all drug-induced cases of AKI, ranked only after antibiotics [68]. The reasons for the association between the use of diuretics and an increased risk of AKI could be interpreted as follows. First, loop diuretics block sodium chloride uptake in the macula densa in a manner that is independent of any effect on sodium and water balance, thereby stimulating the renin-angiotensin-aldosterone system (RAAS) and leading to AKI. Sometimes, AKI is caused by the combined action of diuretics and other drugs, which may include antibiotics, contrast media, ACEI/ARB, and NSAIDs [69]. Another study showed that a triple therapy combination consisting of diuretics with ACEI or ARB and NSAIDs was associated with an increased risk of AKI [70]. However, the high levels of heterogeneity related to these factors cannot be ignored. Furthermore, our subgroup analyses failed to identify the specific sources of such heterogeneity. Therefore, these results should be interpreted with caution. Such heterogeneity may originate from the specific types, duration, and dosage of drugs and their interactions with other drugs. More homogeneous clinical randomized trials should be conducted in patients with sepsis to confirm the specific role of these drugs and their interactions in inducing AKI.

Many studies have confirmed that mechanical ventilation is a risk factor for AKI; our present findings concur with these previous findings [71, 72]. A previous study showed that mechanical ventilation is used in up to 75% of patients in ICU [73]. Our analyses showed that 68% of patients with sepsis who developed AKI also used mechanical ventilation; this proportion is even higher in patients with septic shock and severe sepsis. Therefore, we have to pay special attention to prevent the development of AKI in patients undergoing mechanical ventilation. Hypoxemia, hypercapnia, and excessive positive end-expiratory pressure (PEEP) values during mechanical ventilation are all risk factors for AKI. If there are other risk factors at the same time, AKI is more likely to occur. At present, there is no useful method to prevent or reduce the AKI caused by mechanical ventilation. Some studies have shown that the development of AKI can be reduced by adjusting ventilator parameters, improving hypoxia status as soon as possible, avoiding persistent hypercapnia, and by using smaller PEEP settings. However, a previous meta-analysis showed that invasive MV could be associated with a threefold increase in the odds of AKI in critically ill patients, and tidal volume (Vt) and PEEP settings do not modify this risk [72]. Therefore, future research should focus on strategies that can reduce the risks of AKI induced by mechanical ventilation.

### Limitations

Our study has some limitations that should be considered. First, all of our results were based on unadjusted estimates due to the significant variability of multivariable models across different studies. Therefore, we may have failed to identify independent predictors for AKI in the presence of confounding factors. Secondly, significant heterogeneity was observed for certain risk factors due to varied geographic locations, demographic data, and inconsistent diagnostic criteria for AKI and sepsis. We did not identify the source of this heterogeneity in our subgroup analyses; this issue may have impacted on our results. In addition, due to the small number of studies, heterogeneity and publication bias were not evaluated for certain risk factors.

## Conclusion

Our analyses showed that the most common risk factors for S-AKI were septic shock, hypertension, diabetes mellitus, abdominal infection, a history of smoking, positive blood cultures, the use of vasopressors, and mechanical ventilation. Other risk factors included cardiovascular and coronary artery disease, liver disease, unknown infections, the use of diuretics, the use of ACEI or ARB, gram-negative bacteria infections, and organ transplantation. Despite our rigorous methodology, the inherent limitations of the included studies prevented us from reaching definitive conclusions. However, this article is the first systematic review and meta-analysis to investigate the risk factors for AKI development in patients with sepsis. Our findings may facilitate the development of clinical targeted care strategies for the prevention, detection and management of AKI in patients with sepsis.

## Supplementary information

**Additional file 1.** Checklist.PRISMA Checklist.

**Additional file 2.** Fig. Hypertension-Forest plot, Funnel plot, Sensitivity and Subgroup analysis.

**Additional file 3.** Fig. Diabetes mellitus-Forest plot and Funnel plot.

**Additional file 4.** Fig. Chronic kidney disease-Forest plot, Funnel plot, Sensitivity and Subgroup analysis.

**Additional file 5.** Fig. Cardiovascular Diseases -Forest plot, Funnel plot.

**Additional file 6.** Fig. Liver disease-Forest plot and Sensitivity analysis.

**Additional file 7.** Fig. Coronary artery disease-Forest plot and Funnel plot.

**Additional file 8.** Fig. Pulmonary infection-Forest plot, Funnel plot, Sensitivity and subgroup analysis.

**Additional file 9.** Fig. Abdominal infection-Forest plot, Funnel plot and Sensitivity analysis.

**Additional file 10.** Fig. Unknown source of infection-Forest plot.

**Additional file 11.** Fig. Vasopressors-Forest plot, Funnel plot, Sensitivity and Subgroup analysis.

**Additional file 12.** Fig. Diuretic-Forest plot.

**Additional file 13.** Fig. Sex (male)-Forest plot, Funnel plot, Sensitivity and Subgroup analysis.

**Additional file 14.** Fig. Positive blood culture-Forest plot, Funnel plot and Sensitivity analysis.

**Additional file 15.** Fig. Smoke history-Forest plot, Sensitivity analysis.

**Additional file 16.** Fig. Septic shock-Forest plot and Funnel plot.

**Additional file 17.** Fig.Gram-negative bacteria-Forest plot.

**Additional file 18.** Fig. Organ transplant-Forest plot and Sensitivity analysis.

**Additional file 19.** Fig. Mechanical ventilation-forest plot, Funnel plot, Sensitivity and Subgroup analysis.

## Data Availability

All data generated or analysed during this study are included in this published article [and its seen Additional files and Supplementary materials].

## Abbreviations

AKI: Acute kidney injury
S-AKI: Sepsis-associated acute kidney injury
ARF: Acute renal failure
OR: Odds ratio
CI: Confidence interval
CKD: Chronic kidney disease
KDIGO: Kidney disease improving global outcomes
AKIN: Acute kidney injury network classification
RIFLE: Risk, injury, failure, end stage kidney disease
NSAIDs: Non-steroidal anti-inflammatory drugs
COPD: Chronic obstructive pulmonary disease
ACEI or ARB: Angiotensin converting enzyme inhibitors or Angiotensin receptor blocker
PEEP: Positive end-expiratory pressure
MV: Mechanical ventilation

## References

[1] Alobaidi R, Basu RK, Goldstein SL, Bagshaw SM (2015). Sepsis-associated acute kidney injury. Semin Nephrol.

[2] Bagshaw SM, George C, Bellomo R (2007). Changes in the incidence and outcome for early acute kidney injury in a cohort of Australian intensive care units. Crit Care.

[3] Bagshaw SM, Uchino S, Bellomo R, Morimatsu H, Morgera S, Schetz M, Tan I, Bouman C, Macedo E, Gibney N (2007). Septic acute kidney injury in critically ill patients: clinical characteristics and outcomes. Clin J Am Soc Nephrol.

[4] Bouchard J, Acharya A, Cerda J, Maccariello ER, Madarasu RC, Tolwani AJ, Liang X, Fu P, Liu ZH, Mehta RL (2015). A prospective international multicenter study of AKI in the intensive care unit. Clin J Am Soc Nephrol.

[5] Hsu YC, Hsu CW (2019). Septic acute kidney injury patients in emergency department: the risk factors and its correlation to serum lactate. Am J Emerg Med.

[6] Clifford KM, Dy-Boarman EA, Haase KK, Maxvill K, Pass SE, Alvarez CA (2016). Challenges with diagnosing and managing Sepsis in older adults. Expert Rev Anti Infect Ther.

[7] Rowe TA, McKoy JM (2017). Sepsis in Older Adults. Infect Dis Clin North Am.

[8] Bone RC, Balk RA, Cerra FB (1992). Definitions for sepsis and organ failure and guidelines for the use of innovative therapies in sepsis. The ACCP/SCCM consensus conference committee. American College of Chest Physicians/Society of Critical Care Medicine. Chest.

[9] Levy MM, Fink MP, Marshall JC, Abraham E, Angus D, Cook D, Cohen J (2003). 2001 SCCM/ESICM/ACCP/ATS/SIS international Sepsis definitions conference. Intensive Care Med.

[10] Singer M, Deutschman CS, Seymour CW (2016). The Third International Consensus Definitions for Sepsis and Septic Shock (Sepsis-3). JAMA.

[11] Moher D, Liberati A, Tetzlaff J, Altman DG (2009). Preferred reporting items for systematic reviews and meta-analyses: the PRISMA statement. PLoS Med.

[12] Bu X, Zhang L, Chen P, Wu X (2019). Relation of neutrophil-to-lymphocyte ratio to acute kidney injury in patients with sepsis and septic shock: a retrospective study. Int Immunopharmacol.

[13] Hsu YC, Hsu CW (2019). Septic acute kidney injury patients in emergency department: the risk factors and its correlation to serum lactate. Am J Emerg Med.

[14] Vilander LM, Vaara ST, Donner KM, Lakkisto P, Kaunisto MA, Pettila V (2019). Heme oxygenase-1 repeat polymorphism in septic acute kidney injury. PLoS One.

[15] Xing ZQ, Liu DW, Wang XT, Long Y, Zhang HM, Wang C, Huang W (2019). The value of renal resistance index and urine oxygen pressure for prediction of acute kidney injury in patients with septic shock. Zhonghua Nei Ke Za Zhi.

[16] Moman RN, Ostby SA, Akhoundi A, Kashyap R, Kashani K (2018). Impact of individualized target mean arterial pressure for septic shock resuscitation on the incidence of acute kidney injury: a retrospective cohort study. Ann Intensive Care.

[17] Zhi DY, Lin J, Zhuang HZ (2018). Acute Kidney Injury in Critically Ill Patients with Sepsis: Clinical Characteristics and Outcomes. J Invest Surg.

[18] Zhou X, Liu J, Ji X, Yang X, Duan M (2018). Predictive value of inflammatory markers for acute kidney injury in sepsis patients: analysis of 753 cases in 7 years. Zhonghua Wei Zhong Bing Ji Jiu Yi Xue.

[19] Costa NA, Gut AL, Azevedo PS, Tanni SE, Cunha NB, Fernandes A, Polegato BF, Zornoff L, de Paiva S, Balbi AL, et al. Protein carbonyl concentration as a biomarker for development and mortality in sepsis-associated acute kidney injury. Biosci Rep. 2018;38(1). 10.1042/BSR20171238. 10.1042/BSR20171238 PMC5784177 29263144

[20] Song J, Wu W, He Y, Lin S, Zhu D, Zhong M (2018). Value of the combination of renal resistance index and central venous pressure in the early prediction of sepsis-associated acute kidney injury. J Crit Care.

[21] Hu Q, Ren J, Ren H, Wu J, Wu X, Liu S, Wang G, Gu G, Guo K, Li J (2018). Urinary mitochondrial DNA identifies renal dysfunction and mitochondrial damage in sepsis-associated acute kidney injury. Oxidative Med Cell Longev.

[22] Fatani SH, ALrefai AA, Al-Amodi HS, Kamel HF, Al-Khatieb K, Bader H (2018). Assessment of tumor necrosis factor alpha polymorphism TNF-alpha-238 (rs 361525) as a risk factor for development of acute kidney injury in critically ill patients. Mol Biol Rep.

[23] Gameiro J, Goncalves M, Pereira M (2018). Obesity, acute kidney injury and mortality in patients with sepsis: a cohort analysis. Ren Fail.

[24] Katayama S, Nunomiya S, Koyama K, Wada M, Koinuma T, Goto Y, Tonai K, Shima J (2017). Markers of acute kidney injury in patients with sepsis: the role of soluble thrombomodulin. Crit Care.

[25] Vilander LM, Kaunisto MA, Vaara ST, Pettila V (2017). Genetic variants in SERPINA4 and SERPINA5, but not BCL2 and SIK3 are associated with acute kidney injury in critically ill patients with septic shock. Crit Care.

[26] Suberviola B, Rodrigo E, Gonzalez-Castro A, Serrano M, Heras M, Castellanos-Ortega A (2017). Association between exposure to angiotensin-converting enzyme inhibitors and angiotensin receptor blockers prior to septic shock and acute kidney injury. Med Int.

[27] Fisher J, Russell JA, Bentzer P, Parsons D, Secchia S, Morgelin M, Walley KR, Boyd JH, Linder A (2017). Heparin-binding protein (HBP): a causative marker and potential target for heparin treatment of human sepsis-associated acute kidney injury. Shock.

[28] Perez-Fernandez X, Sabater-Riera J, Ballus-Noguera J, Cardenas-Campos P, Moreno-Gonzalez G, Alonso-Juste V, Corral-Velez V, Gutierrez-Arambula D, Gumucio-Sanguino V, Betbese-Roig A (2017). No impact of surviving sepsis campaign care bundles in reducing sepsis-associated acute kidney injury. Clin Nephrol.

[29] Pereira M, Rodrigues N, Godinho I, Gameiro J, Neves M, Gouveia J, Costa ESZ, Lopes JA (2017). Acute kidney injury in patients with severe sepsis or septic shock: a comparison between the 'Risk, injury, failure, loss of kidney function, end-stage kidney disease’ (RIFLE), acute kidney injury network (AKIN) and kidney disease: improving global outcomes (KDIGO) classifications. Clin Kidney J.

[30] Panich T, Chancharoenthana W, Somparn P, Issara-Amphorn J, Hirankarn N, Leelahavanichkul A (2017). Urinary exosomal activating transcriptional factor 3 as the early diagnostic biomarker for sepsis-associated acute kidney injury. BMC Nephrol.

[31] Su CM, Cheng HH, Hung CW (2016). The value of serial serum cell adhesion molecules in predicting acute kidney injury after severe sepsis in adults. Clin Chim Acta.

[32] Yilmaz H, Cakmak M, Inan O, Darcin T, Akcay A (2015). Can neutrophil-lymphocyte ratio be independent risk factor for predicting acute kidney injury in patients with severe sepsis?. Ren Fail.

[33] Medeiros P, Nga HS, Menezes P, Bridi R, Balbi A, Ponce D (2015). Acute kidney injury in septic patients admitted to emergency clinical room: risk factors and outcome. Clin Exp Nephrol.

[34] Dai X, Zeng Z, Fu C, Zhang S, Cai Y, Chen Z (2015). Diagnostic value of neutrophil gelatinase-associated lipocalin, cystatin C, and soluble triggering receptor expressed on myeloid cells-1 in critically ill patients with sepsis-associated acute kidney injury. Crit Care.

[35] Sood M, Mandelzweig K, Rigatto C, Tangri N, Komenda P, Martinka G, Arabi Y, Keenan S, Kumar A, Kumar A (2014). Non-pulmonary infections but not specific pathogens are associated with increased risk of AKI in septic shock. Intensive Care Med.

[36] Peng Q, Zhang L, Ai Y, Zhang L (2014). Epidemiology of acute kidney injury in intensive care septic patients based on the KDIGO guidelines. Chin Med J.

[37] Patschan D, Heeg M, Brier M, Brandhorst G, Schneider S, Muller GA (2014). Koziolek MJ.CD4+ lymphocyte adenosine triphosphate--a new marker in sepsis with acute kidney injury?. BMC Nephrol.

[38] Tu Y, Wang H, Sun R, Ni Y, Ma L, Xv F, Hu X, Jiang L, Wu A, Chen X (2014). Urinary netrin-1 and KIM-1 as early biomarkers for septic acute kidney injury. Ren Fail.

[39] Fan H, Zhao Y, Zhu JH, Song FC (2014). Urine neutrophil gelatinase-associated lipocalin in septic patients with and without acute kidney injury. Ren Fail.

[40] Cho E, Lee JH, Lim HJ, Oh SW, Jo SK, Cho WY, Kim HK, Lee SY (2014). Soluble CD25 is increased in patients with sepsis-associated acute kidney injury. Nephrology (Carlton).

[41] Terzi I, Papaioannou V, Papanas N, Dragoumanis C, Petala A, Theodorou V, Gioka T, Vargemezis V, Maltezos E, Pneumatikos I (2014). Alpha1-microglobulin as an early biomarker of sepsis-associated acute kidney injury: a prospective cohort study. Hippokratia.

[42] Poukkanen M, Wilkman E, Vaara ST, Pettila V, Kaukonen KM, Korhonen AM, Uusaro A, Hovilehto S, Inkinen O, Laru-Sompa R (2013). Hemodynamic variables and progression of acute kidney injury in critically ill patients with severe sepsis: data from the prospective observational FINNAKI study. Crit Care.

[43] Legrand M, Dupuis C, Simon C, Gayat E, Mateo J, Lukaszewicz AC, Payen D (2013). Association between systemic hemodynamics and septic acute kidney injury in critically ill patients: a retrospective observational study. Crit Care.

[44] Cardinal-Fernandez P, Ferruelo A, El-Assar M, Santiago C, Gomez-Gallego F, Martin-Pellicer A, Frutos-Vivar F, Penuelas O, Nin N, Esteban A (2013). Genetic predisposition to acute kidney injury induced by severe sepsis. J Crit Care.

[45] de Geus HR, Fortrie G, Betjes MG, van Schaik RH, Groeneveld AB (2013). Time of injury affects urinary biomarker predictive values for acute kidney injury in critically ill, non-septic patients. BMC Nephrol.

[46] Katagiri D, Doi K, Matsubara T, Negishi K, Hamasaki Y, Nakamura K, Ishii T, Yahagi N, Noiri E (2013). New biomarker panel of plasma neutrophil gelatinase-associated lipocalin and endotoxin activity assay for detecting sepsis in acute kidney injury. J Crit Care.

[47] Aydogdu M, Gursel G, Sancak B, Yeni S, Sari G, Tasyurek S, Turk M, Yuksel S, Senes M, Ozis TN (2013). The use of plasma and urine neutrophil gelatinase associated lipocalin (NGAL) and Cystatin C in early diagnosis of septic acute kidney injury in critically ill patients. Dis Markers.

[48] Suh SH, Kim CS, Choi JS, Bae EH, Ma SK, Kim SW (2013). Acute kidney injury in patients with sepsis and septic shock: risk factors and clinical outcomes. Yonsei Med J.

[49] Poukkanen M, Vaara ST, Pettila V, Kaukonen KM, Korhonen AM, Hovilehto S, Inkinen O, Laru-Sompa R, Kaminski T, Reinikainen M (2013). Acute kidney injury in patients with severe sepsis in Finnish intensive care units. Acta Anaesthesiol Scand.

[50] Zhao N, Tian HH, Li Z (2013). Risk factors and early diagnosis of acute kidney injury in patients with sepsis. Zhonghua Wei Zhong Bing Ji Jiu Yi Xue.

[51] Payen D, Lukaszewicz AC, Legrand M, Gayat E, Faivre V, Megarbane B, Azoulay E, Fieux F, Charron D, Loiseau P (2012). A multicentre study of acute kidney injury in severe sepsis and septic shock: association with inflammatory phenotype and HLA genotype. PLoS One.

[52] Frank AJ, Sheu CC, Zhao Y, Chen F, Su L, Gong MN, Bajwa E, Thompson BT, Christiani DC (2012). BCL2 genetic variants are associated with acute kidney injury in septic shock*. Crit Care Med.

[53] Plataki M, Kashani K, Cabello-Garza J, Maldonado F, Kashyap R, Kor DJ, Gajic O, Cartin-Ceba R (2011). Predictors of acute kidney injury in septic shock patients: an observational cohort study. Clin J Am Soc Nephrol.

[54] Martensson J, Bell M, Oldner A, Xu S, Venge P, Martling CR (2010). Neutrophil gelatinase-associated lipocalin in adult septic patients with and without acute kidney injury. Intensive Care Med.

[55] Yang RL, Wang XT, Liu DW (2009). The hemodynamic characteristic and prognosis significance of acute kidney injury caused by septic shock. Zhonghua Nei Ke Za Zhi.

[56] Lopes JA, Jorge S, Resina C, Santos C, Pereira A, Neves J, Antunes F, Prata MM (2009). Acute kidney injury in patients with sepsis: a contemporary analysis. Int J Infect Dis.

[57] Bagshaw SM, Lapinsky S, Dial S, Arabi Y, Dodek P, Wood G, Ellis P, Guzman J, Marshall J, Parrillo JE (2009). Acute kidney injury in septic shock: clinical outcomes and impact of duration of hypotension prior to initiation of antimicrobial therapy. Intensive Care Med.

[58] Bagshaw SM, George C, Bellomo R (2008). Early acute kidney injury and sepsis: a multicentre evaluation. Crit Care.

[59] Rowe TA, McKoy JM (2017). Sepsis in Older Adults. Infect Dis Clin N Am.

[60] Girman CJ, Kou TD, Brodovicz K, Alexander CM, O'Neill EA, Engel S, Williams-Herman DE, Katz L (2012). Risk of acute renal failure in patients with type 2 diabetes mellitus. Diabet Med.

[61] Sathananthan M, Sathananthan A, Jeganathan N. Characteristics and outcomes of patients with and without type 2 diabetes mellitus and pulmonary Sepsis. J Intensive Care Med. 2019:1585820106. 10.1177/0885066619833910. 10.1177/0885066619833910 30841774

[62] Greenberg N, Roberts WL, Bachmann LM, Wright EC, Dalton RN, Zakowski JJ, Miller WG (2012). Specificity characteristics of 7 commercial creatinine measurement procedures by enzymatic and Jaffe method principles. Clin Chem.

[63] James MT, Grams ME, Woodward M, Elley CR, Green JA, Wheeler DC, de Jong P, Gansevoort RT, Levey AS, Warnock DG (2015). A Meta-analysis of the Association of Estimated GFR, Albuminuria, Diabetes Mellitus, and Hypertension With Acute Kidney Injury. Am J Kidney Dis.

[64] Hsu RK, Hsu CY (2016). The role of acute kidney injury in chronic kidney disease. Semin Nephrol.

[65] He L, Wei Q, Liu J, Yi M, Liu Y, Liu H, Sun L, Peng Y, Liu F, Venkatachalam MA (2017). AKI on CKD: heightened injury, suppressed repair, and the underlying mechanisms. Kidney Int.

[66] Grams ME, Sang Y, Ballew SH, Gansevoort RT, Kimm H, Kovesdy CP, Naimark D, Oien C, Smith DH, Coresh J (2015). A Meta-analysis of the Association of Estimated GFR, albuminuria, age, race, and sex with acute kidney injury. Am J Kidney Dis.

[67] Mansfield KE, Nitsch D, Smeeth L (2016). Prescription of renin-angiotensin system blockers and risk of acute kidney injury a population-based cohort study. BMJ Open.

[68] Che ML, Yan YC, Zhang Y (2009). Analysis of drug-induced acute renal failure in Shanghai. Zhonghua Yi Xue Za Zhi.

[69] Wu X, Zhang W, Ren H, Chen X, Xie J, Chen N (2014). Diuretics associated acute kidney injury: clinical and pathological analysis. Ren Fail.

[70] Camin RM, Cols M, Chevarria JL (2015). Acute kidney injury secondary to a combination of renin-angiotensin system inhibitors, diuretics and NSAIDS: "the triple whammy". Nefrologia.

[71] Koyner JL, Murray PT (2010). Mechanical ventilation and the kidney. Blood Purif.

[72] van den Akker JP, Egal M, Groeneveld AB (2013). Invasive mechanical ventilation as a risk factor for acute kidney injury in the critically ill: a systematic review and meta-analysis. Crit Care.

[73] Wunsch H, Angus DC, Harrison DA, Linde-Zwirble WT, Rowan KM (2011). Comparison of medical admissions to intensive care units in the United States and United Kingdom. Am J Respir Crit Care Med.

