# Supplementary material for: Rates, predictors, and mortality of sepsis-associated acute kidney injury: a systematic review and meta-analysis
Source: BMC Nephrol. 2020 Jul 31;21:318. doi: 10.1186/s12882-020-01974-8 (PMC7393862; doi:10.1186/s12882-020-01974-8)

Fig1 Hypertension-Forest map


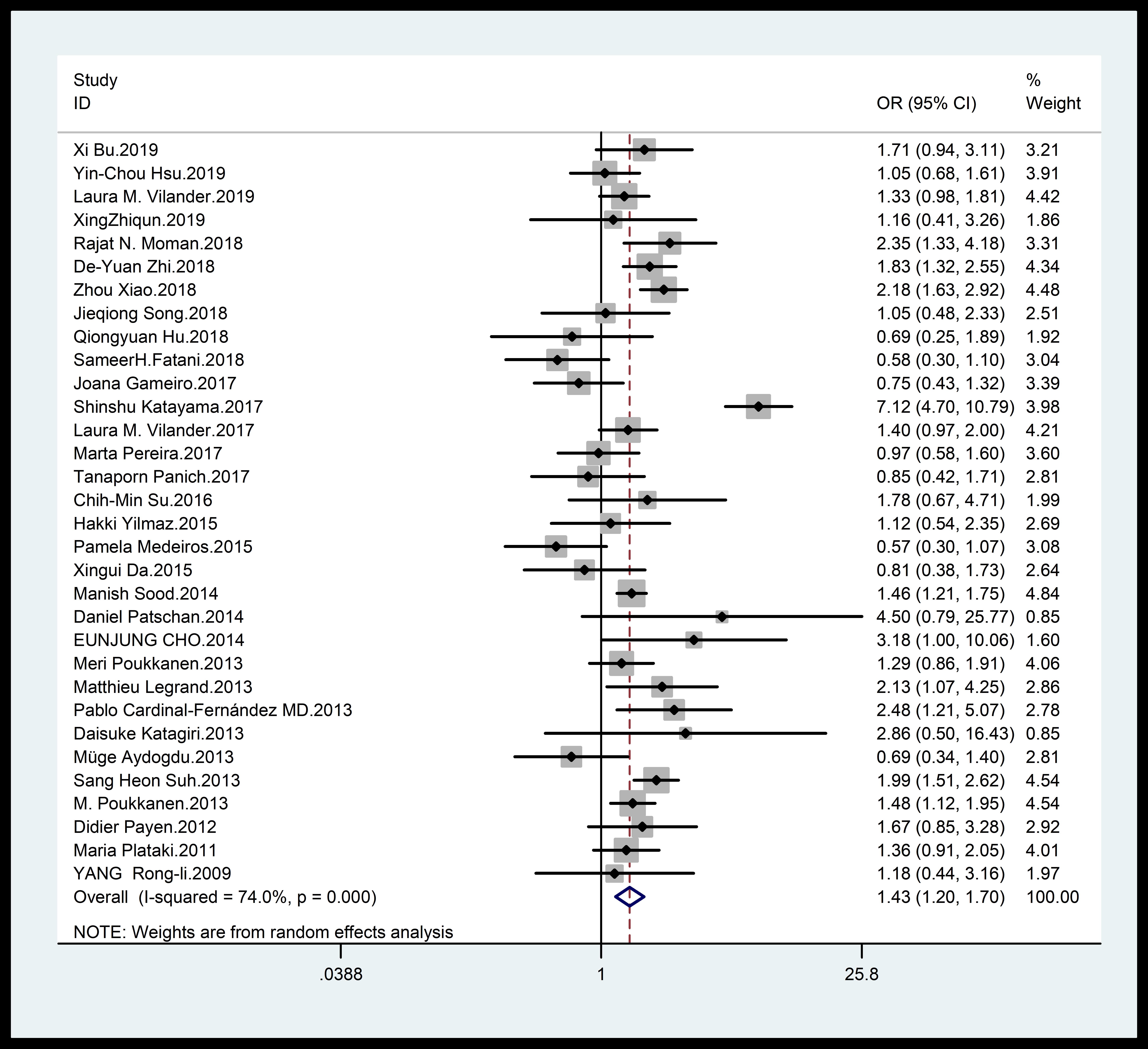


Fig2 Hypertension-Funnel plot


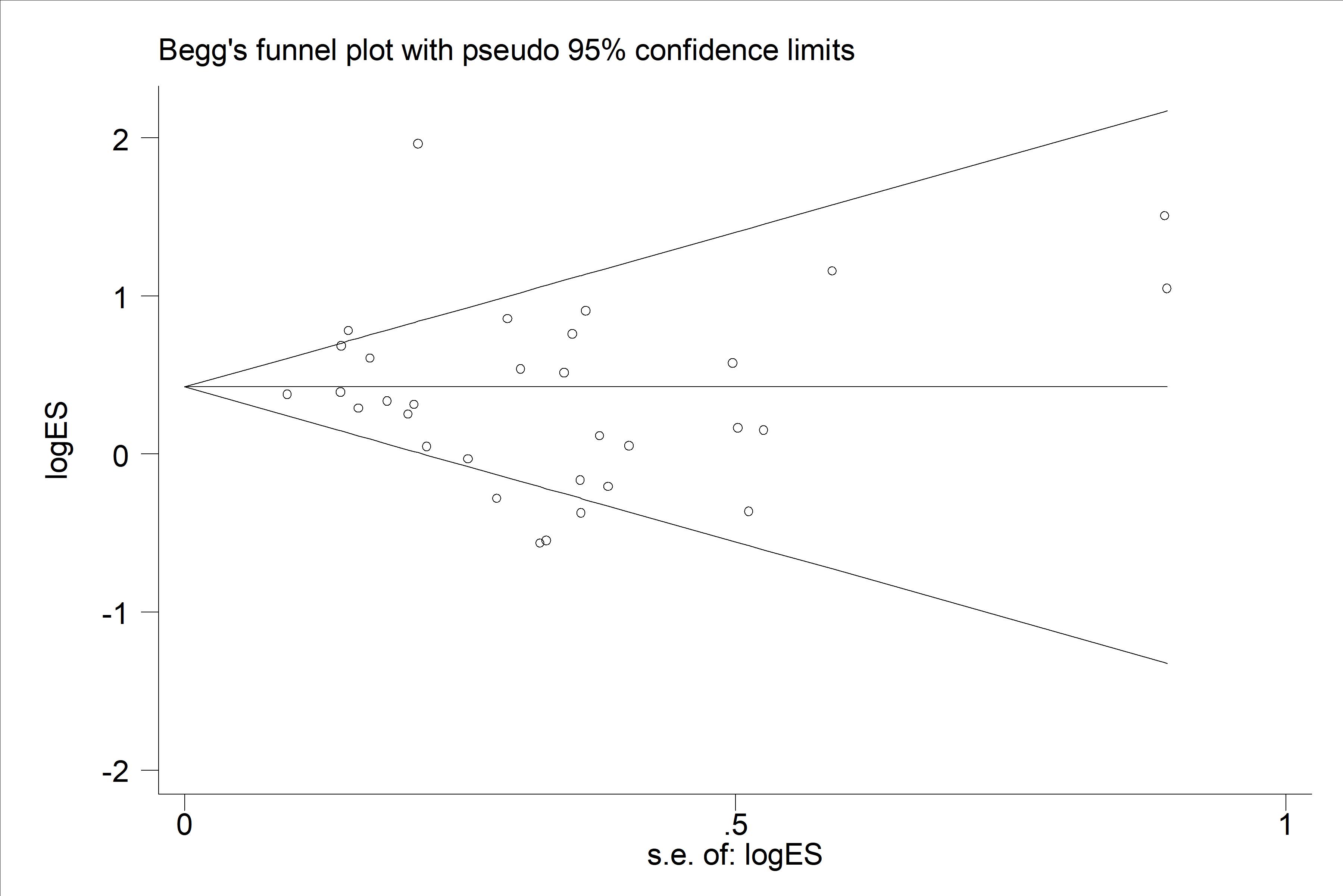


Fig3 Hypertension-Sensitivity analysis


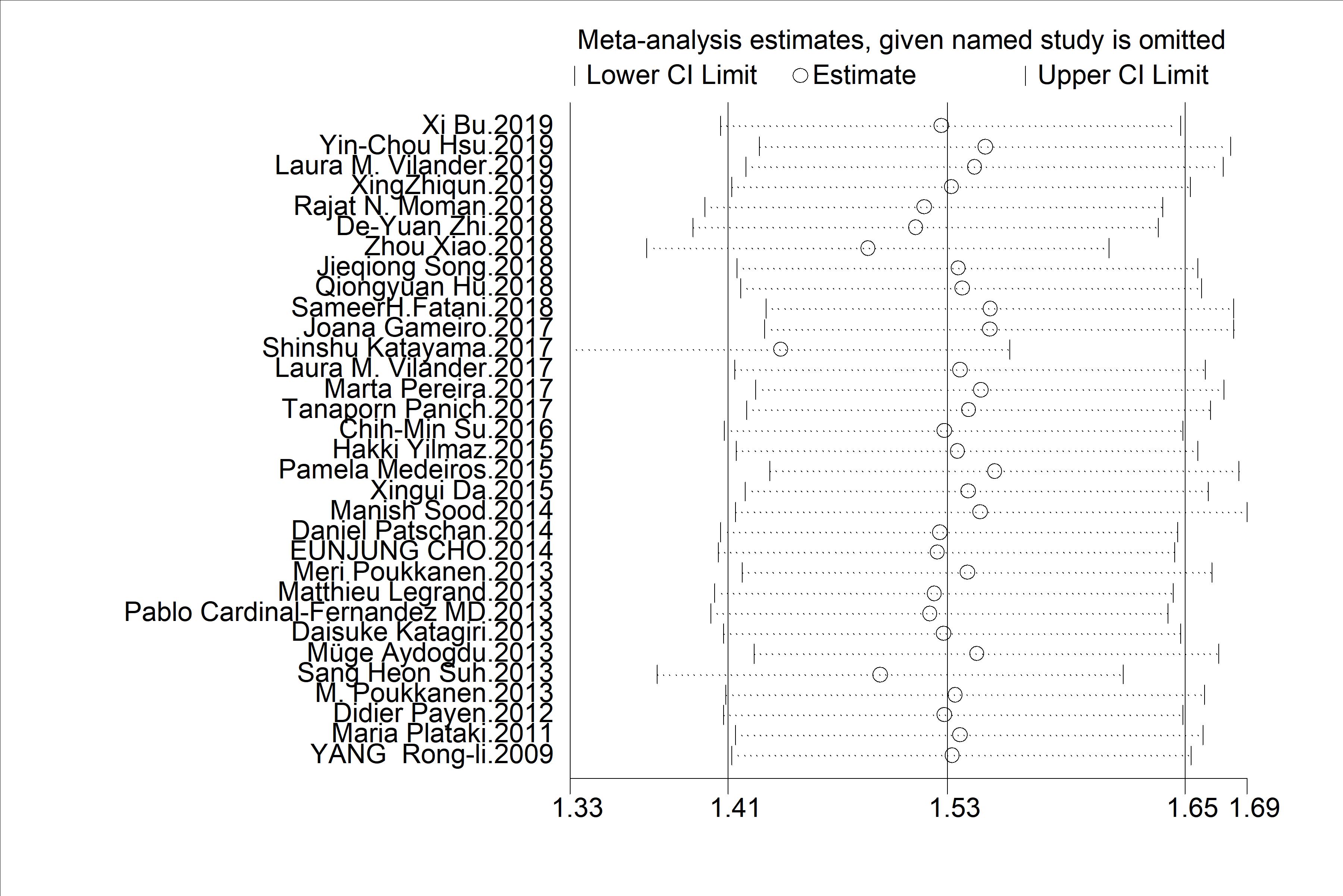


Fig4 Hypertension-Subgroup analysis


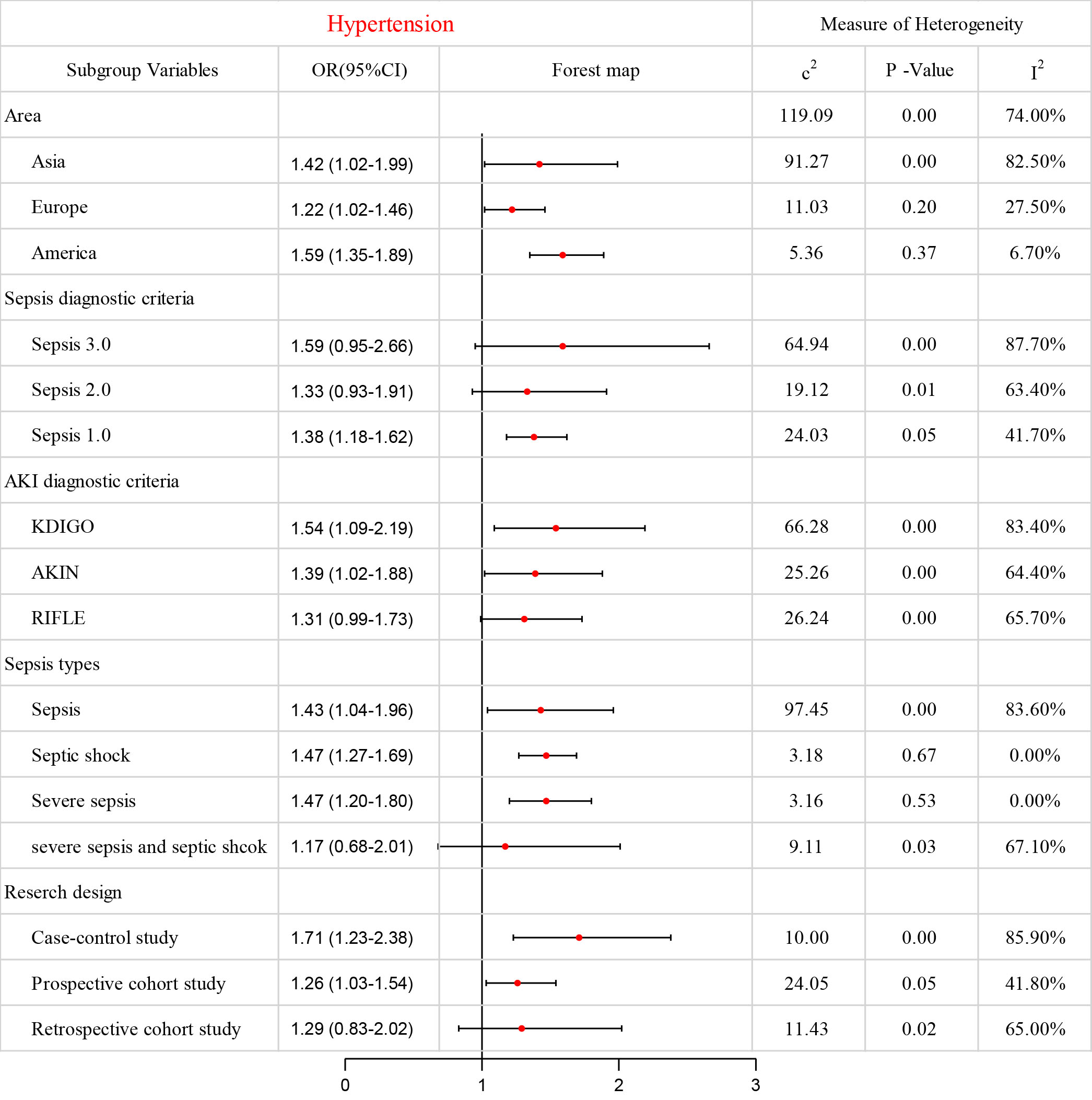

Supplement: Supplementary file 2 — Additional file 2. Fig. Hypertension-Forest plot, Funnel plot, Sensitivity and Subgroup analysis. [file 12882_2020_1974_MOESM2_ESM.doc]
