# Supplementary figures and images for: Rates, predictors, and mortality of sepsis-associated acute kidney injury: a systematic review and meta-analysis
Source: BMC Nephrol. 2020 Jul 31;21:318. doi: 10.1186/s12882-020-01974-8 (PMC7393862; doi:10.1186/s12882-020-01974-8)

Fig1 Cardiovascular Diseases -Forest map


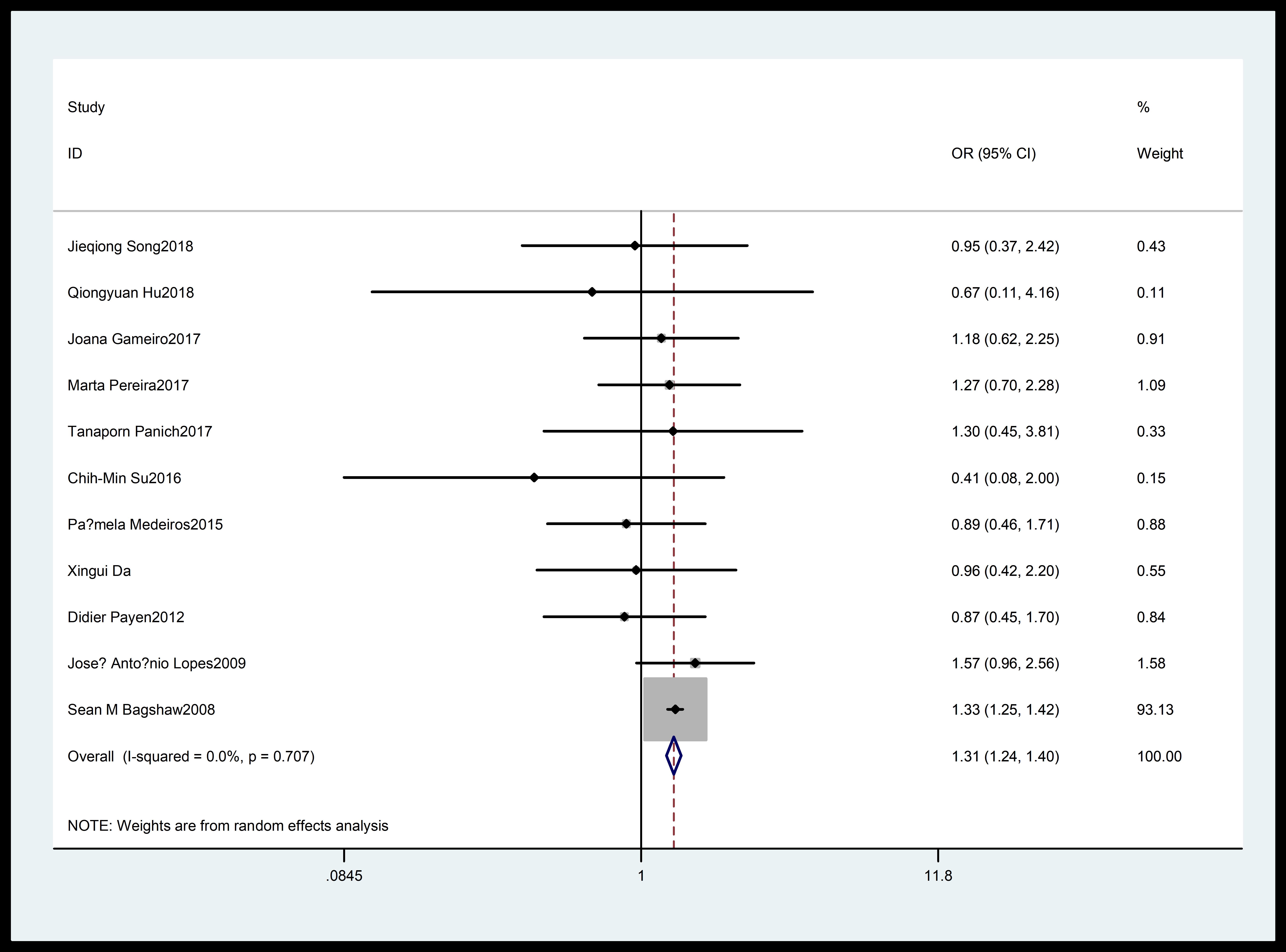


Fig2 Cardiovascular Diseases -Funnel plot


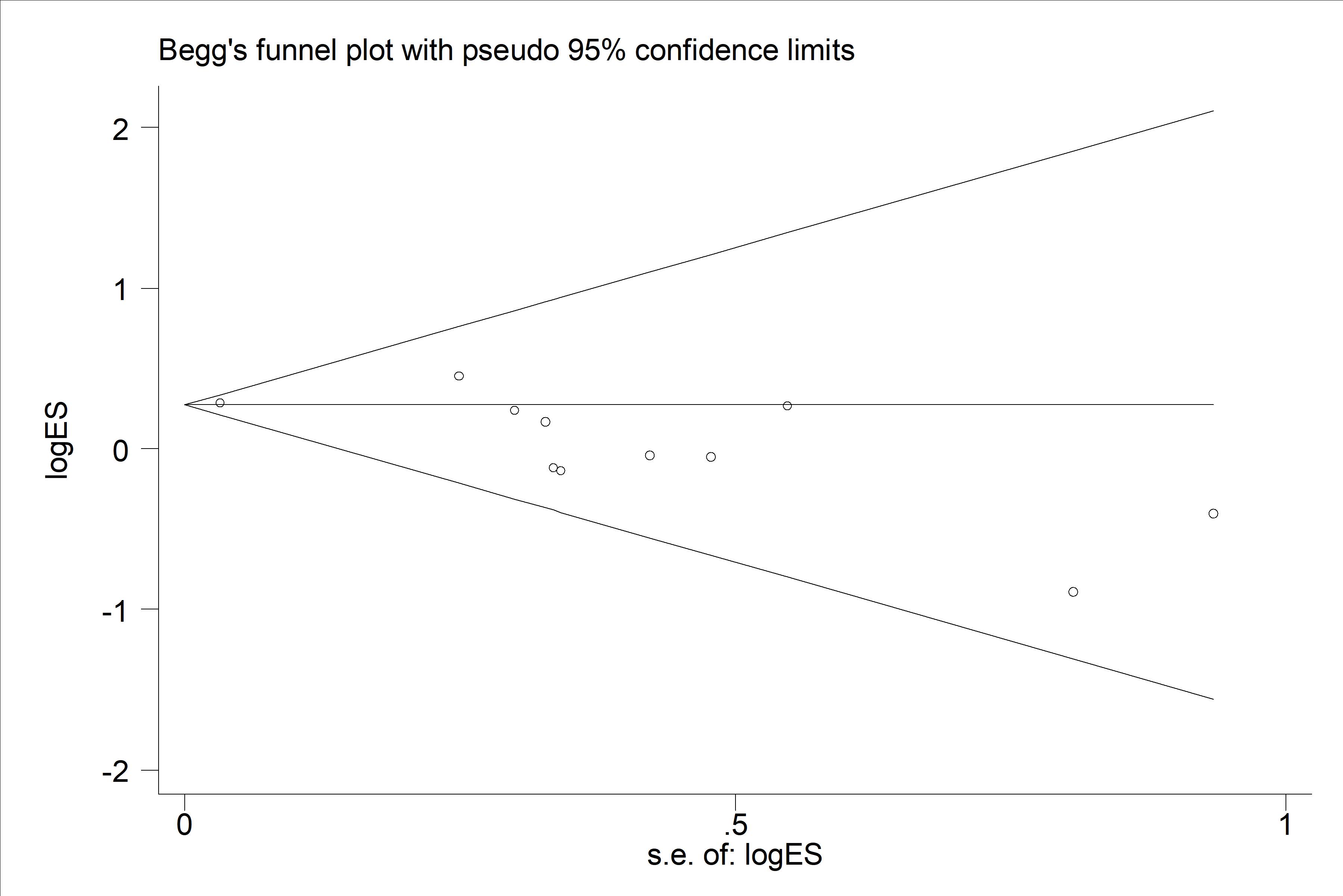

Supplement: Supplementary file 5 — Additional file 5. Fig. Cardiovascular Diseases -Forest plot, Funnel plot. [file 12882_2020_1974_MOESM5_ESM.doc]

Fig1 Coronary artery disease-Forest map


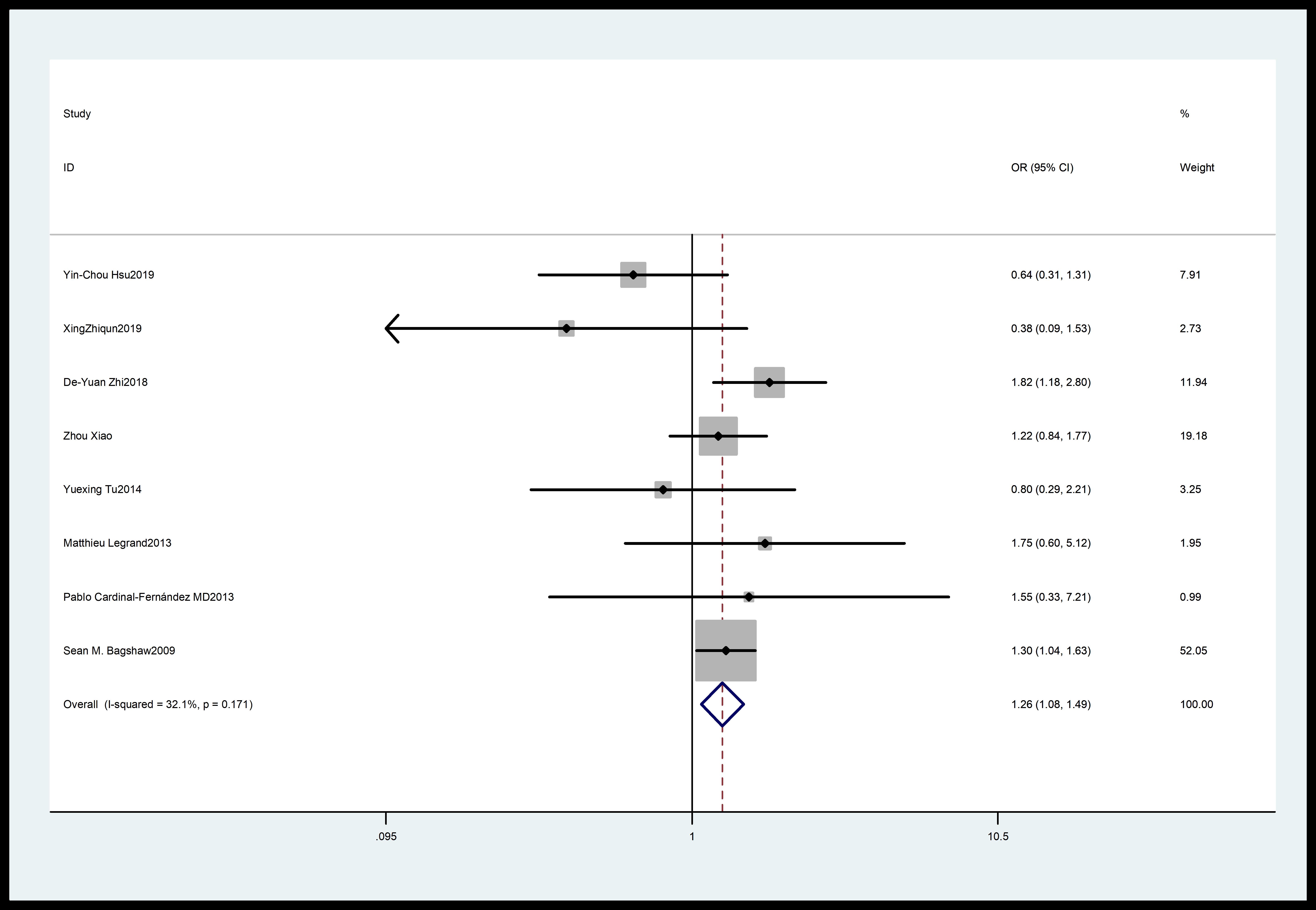


Fig2 Coronary artery disease-Funnel plot


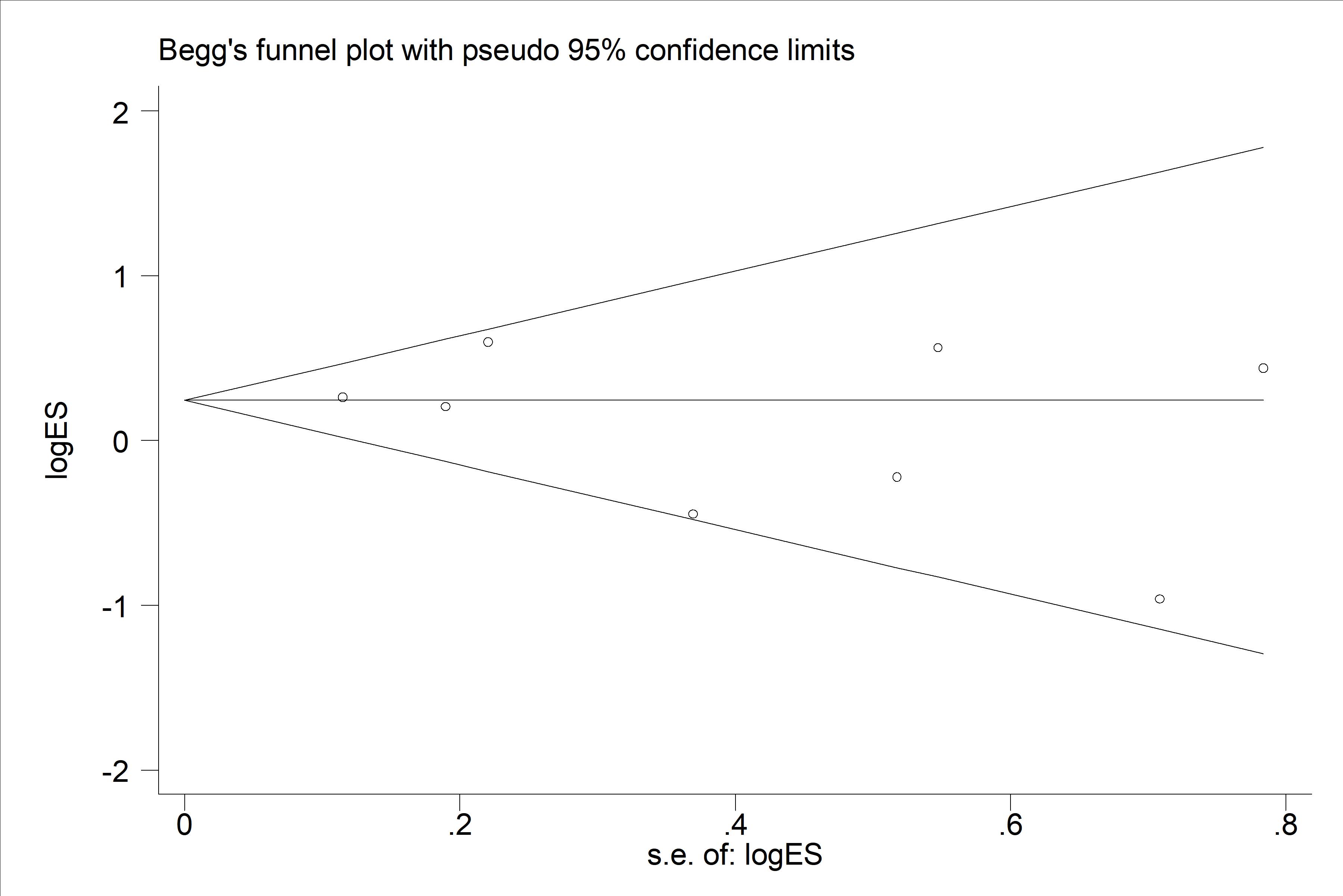

Supplement: Supplementary file 7 — Additional file 7. Fig. Coronary artery disease-Forest plot and Funnel plot. [file 12882_2020_1974_MOESM7_ESM.doc]

Fig1 Use of diuretics-Forest plot(Fixed effect)


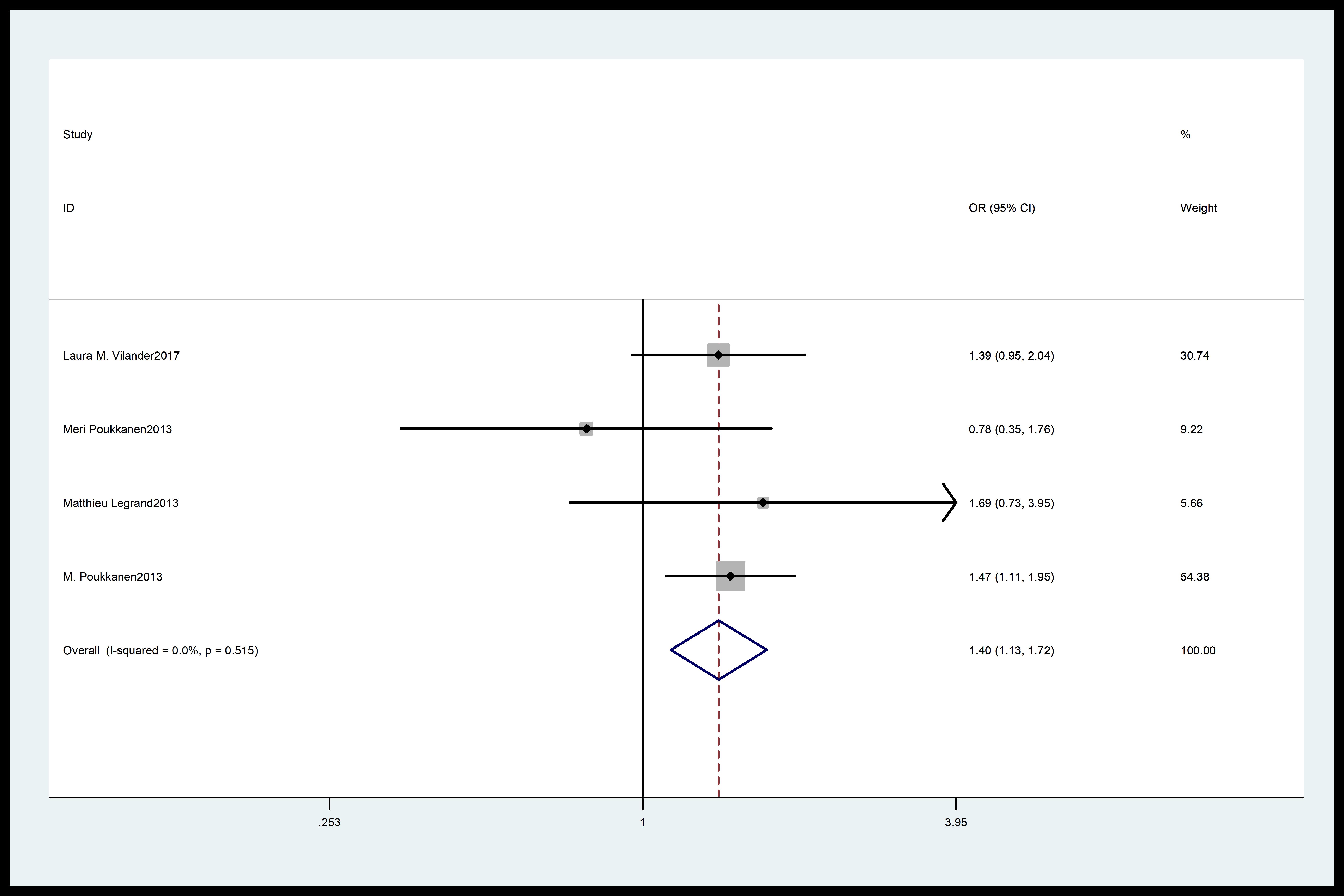


Fig2 Use of diuretics-Forest plot(random effect)


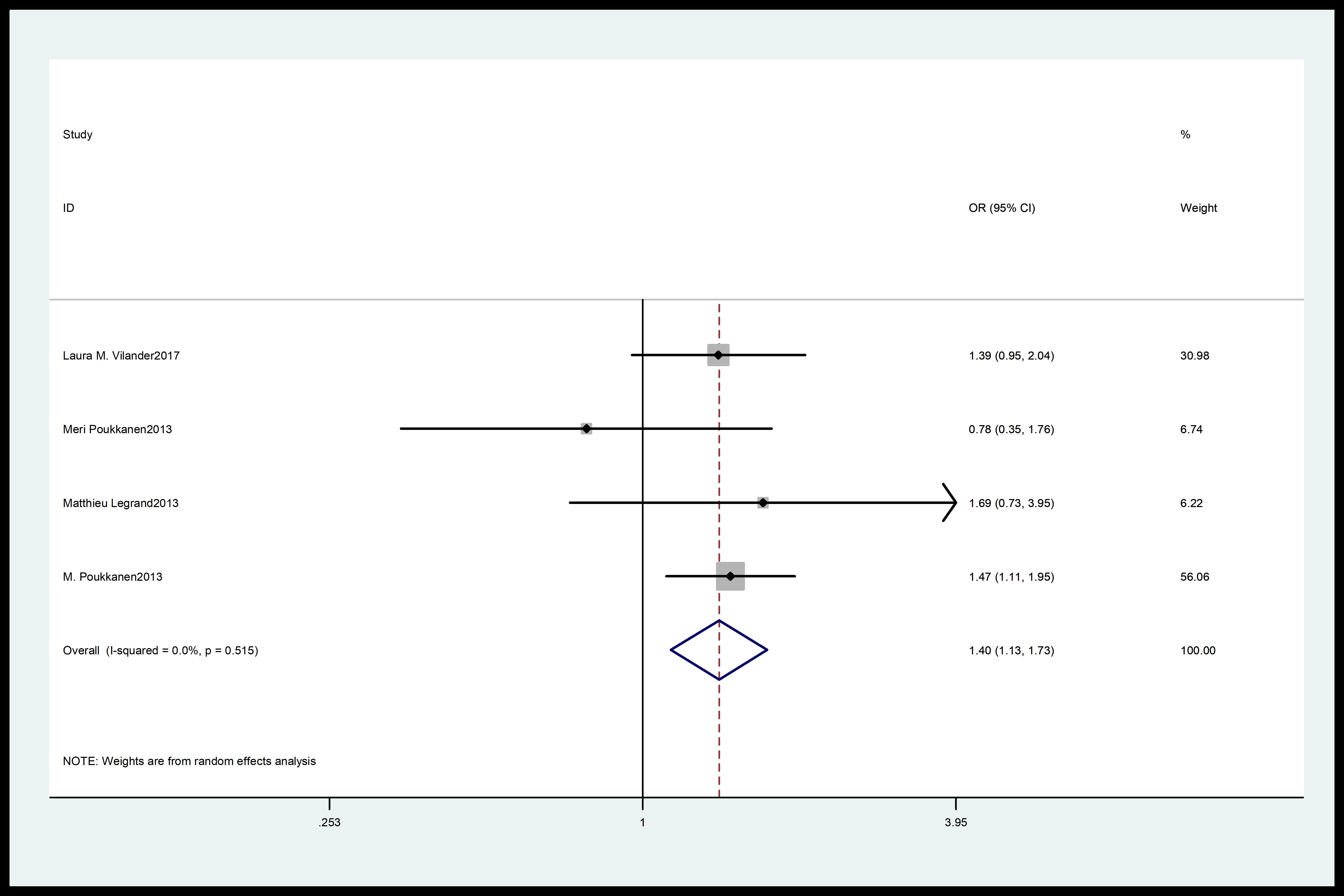

Supplement: Supplementary file 12 — Additional file 12. Fig. Diuretic-Forest plot. [file 12882_2020_1974_MOESM12_ESM.doc]

Fig1 Smoke history-Forest map


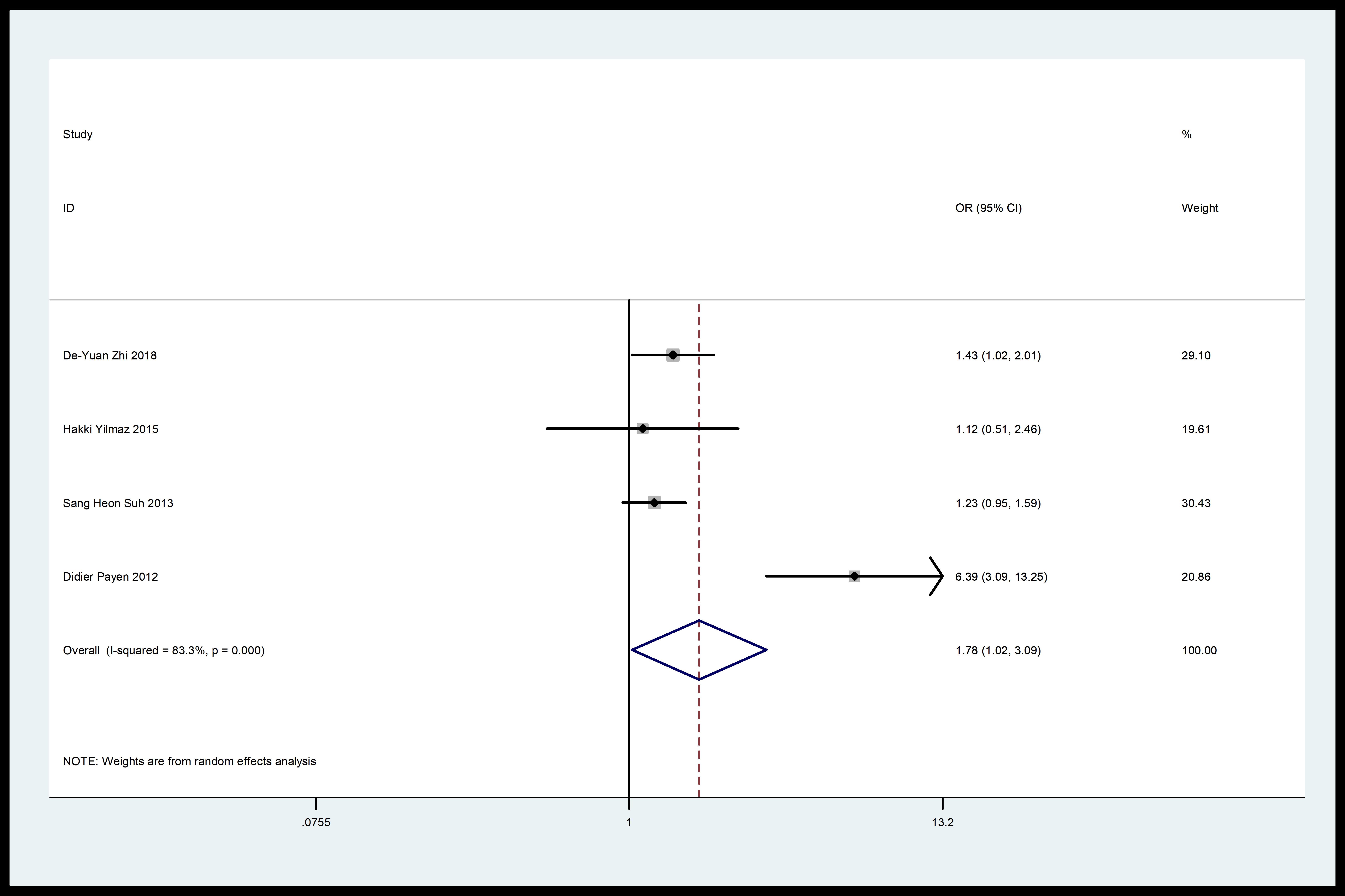


Fig2 Smoke history-Sensitivity analysis


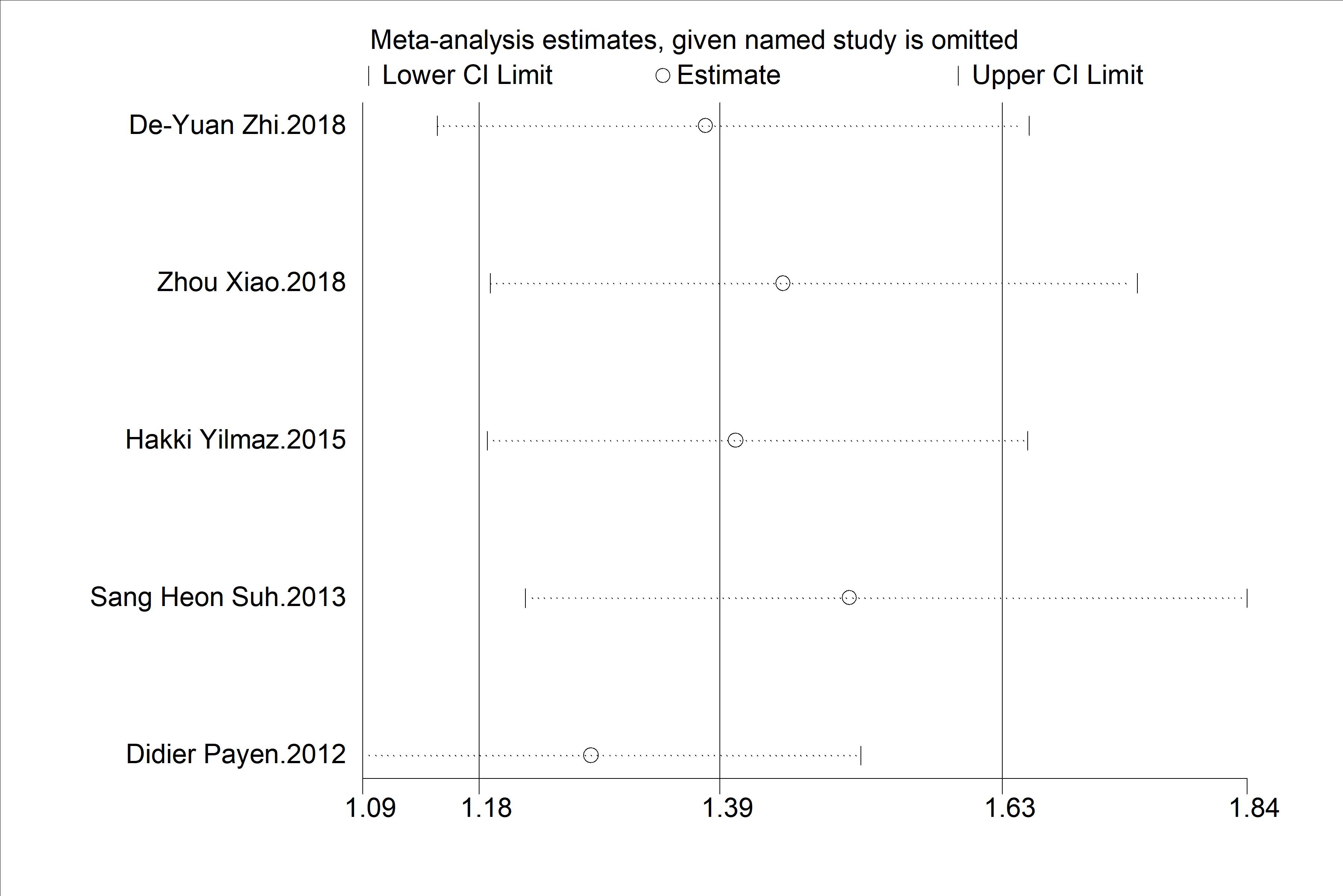

Supplement: Supplementary file 15 — Additional file 15. Fig. Smoke history-Forest plot, Sensitivity analysis. [file 12882_2020_1974_MOESM15_ESM.doc]

Fig1 Gram-negative bacteria-Forest map(fixed effect)


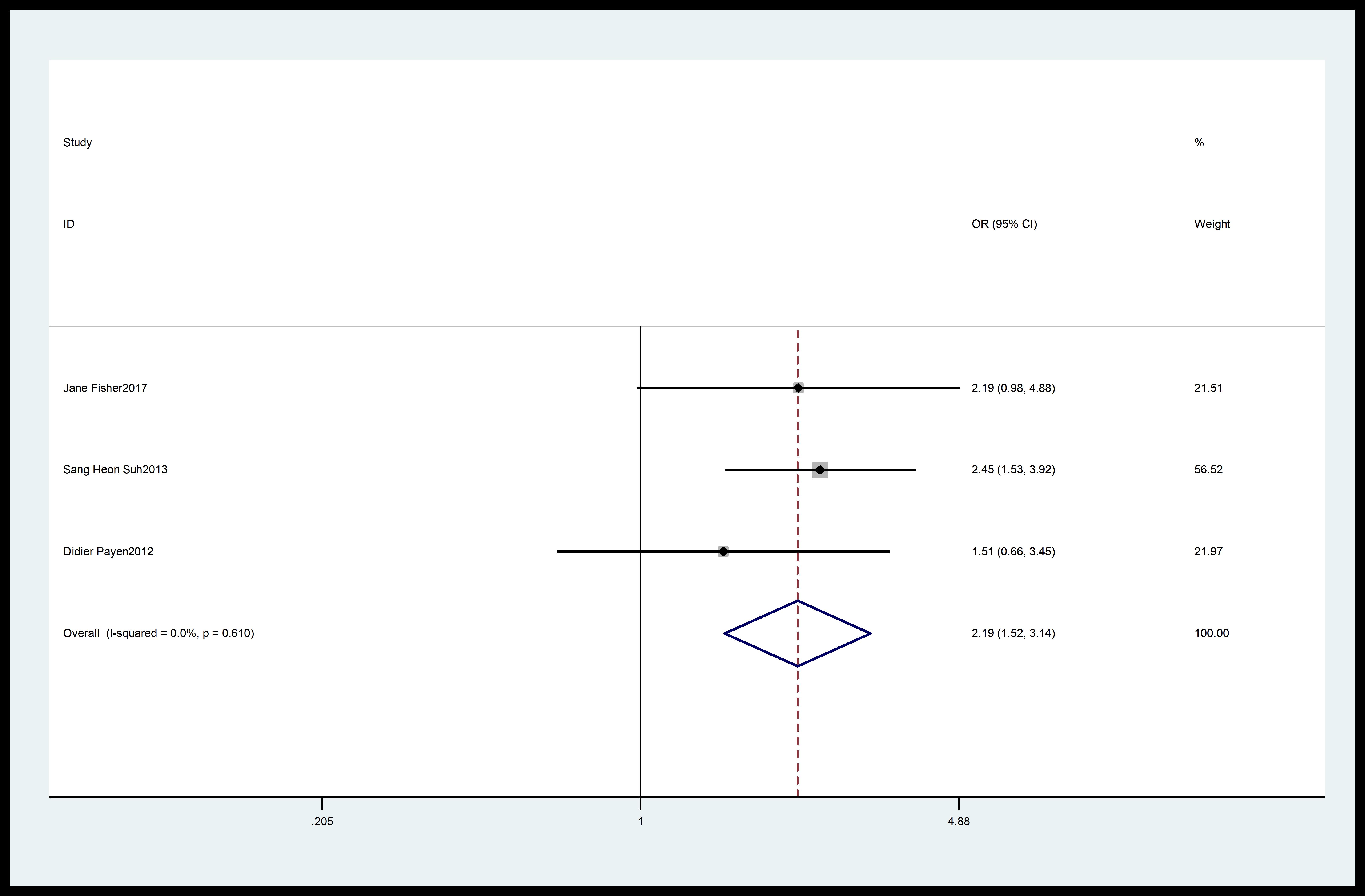

Supplement: Supplementary file 17 — Additional file 17. Fig.Gram-negative bacteria-Forest plot. [file 12882_2020_1974_MOESM17_ESM.doc]

Fig1 Organ transplant-Forest plot


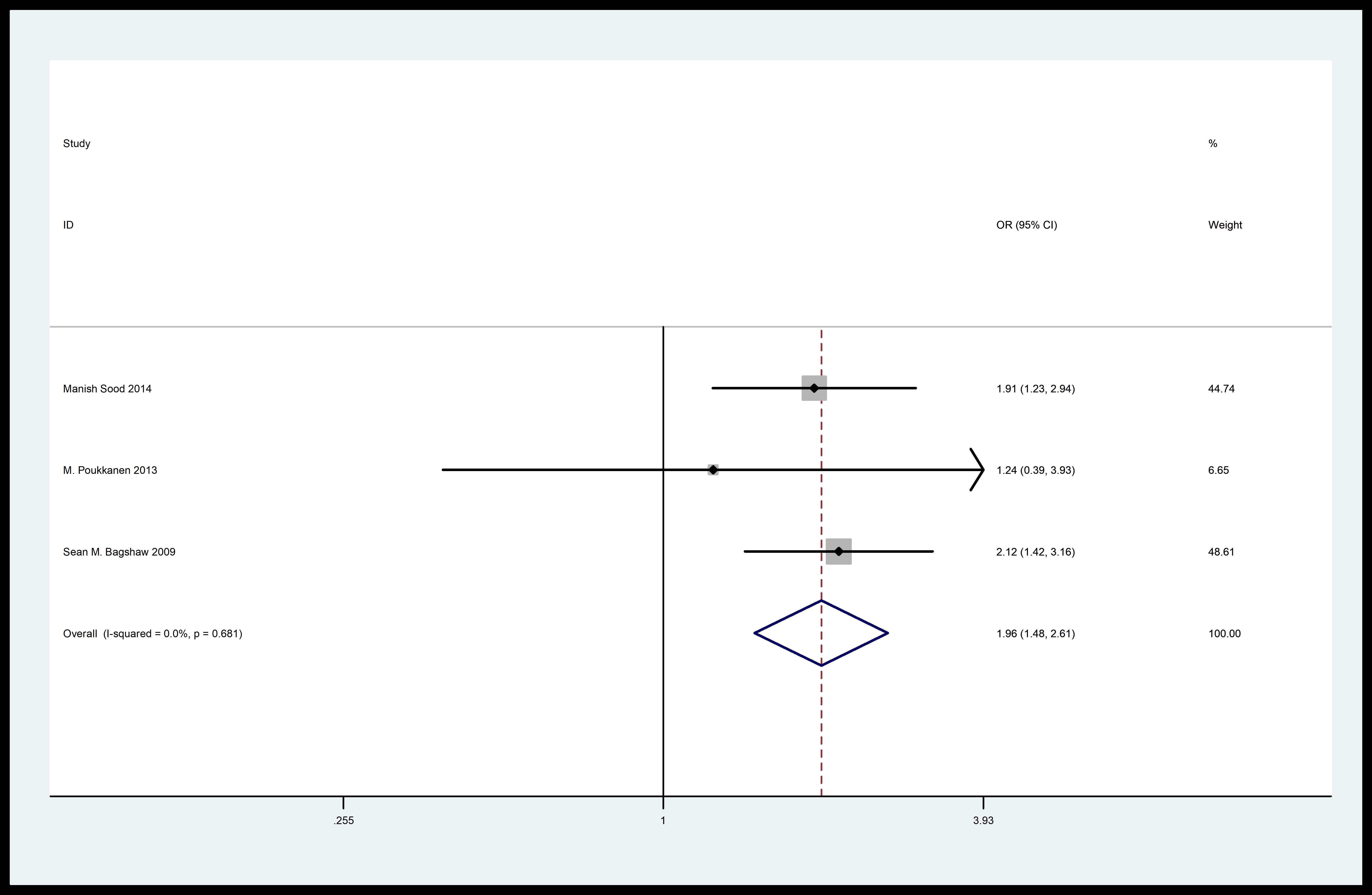


Fig2 Organ transplant-Sensitivity analysis


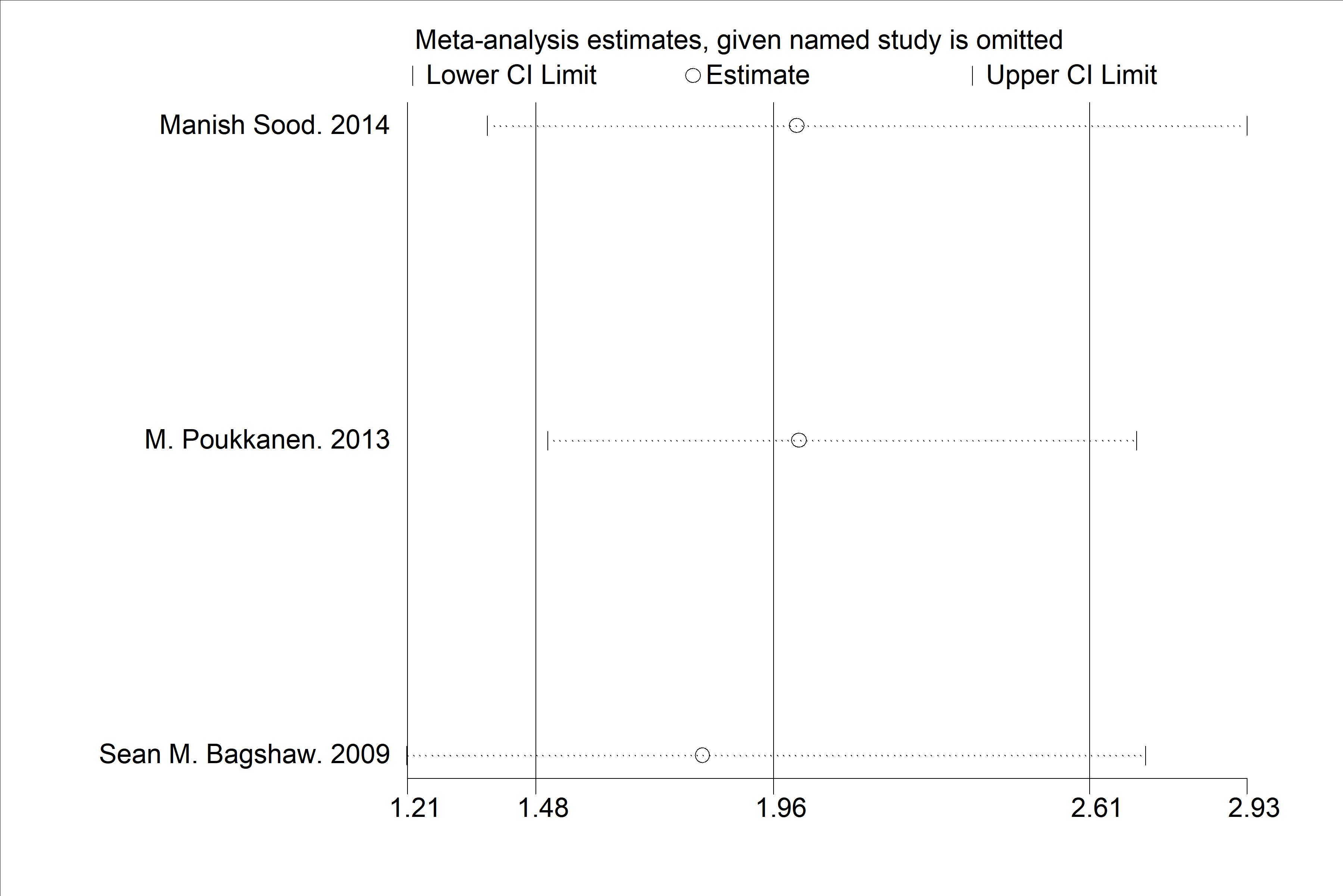

Supplement: Supplementary file 18 — Additional file 18. Fig. Organ transplant-Forest plot and Sensitivity analysis. [file 12882_2020_1974_MOESM18_ESM.doc]
