# Supplementary material for: Rates, predictors, and mortality of sepsis-associated acute kidney injury: a systematic review and meta-analysis
Source: BMC Nephrol. 2020 Jul 31;21:318. doi: 10.1186/s12882-020-01974-8 (PMC7393862; doi:10.1186/s12882-020-01974-8)

Fig1 Liver disease-Forest map(Fixed effect)
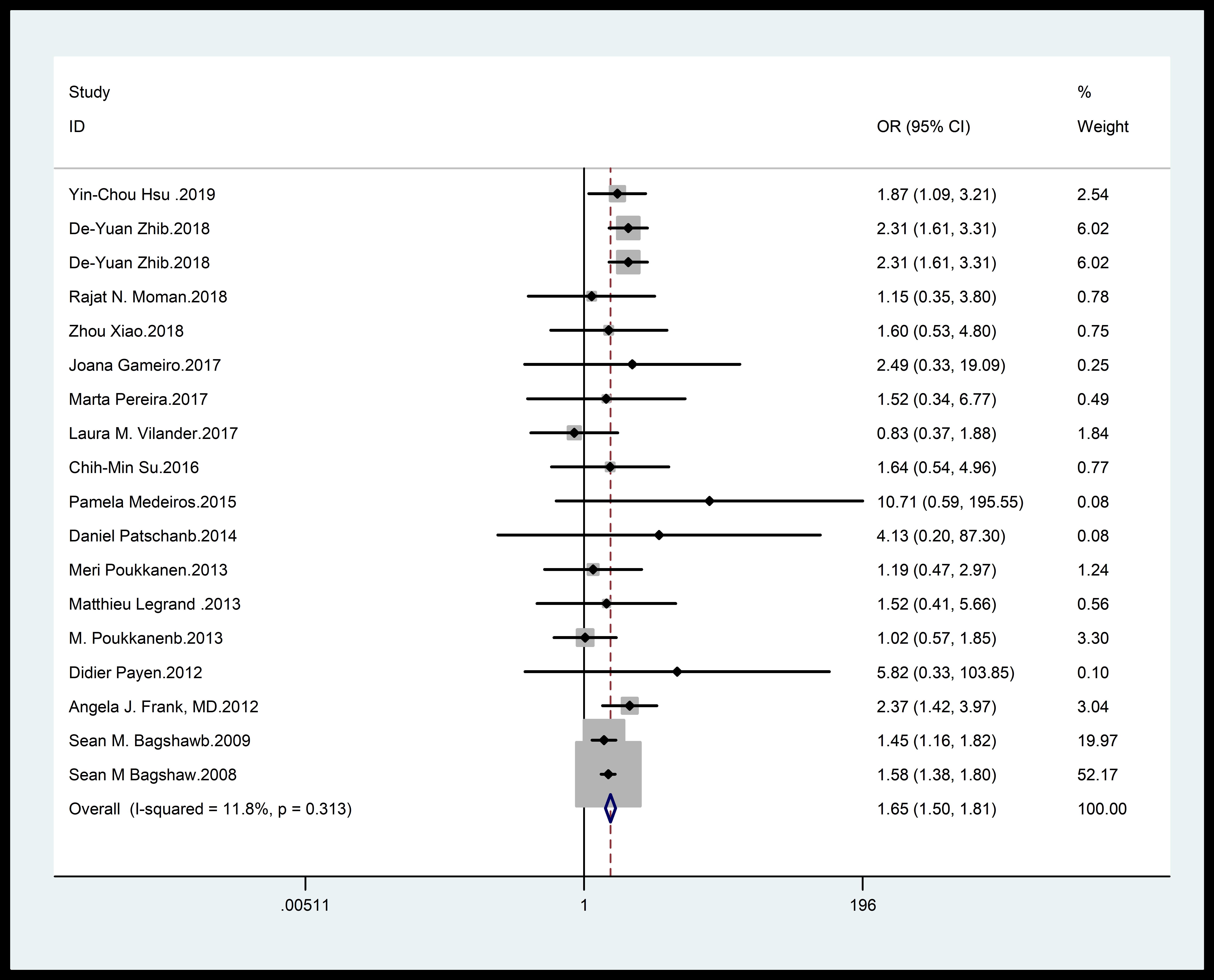


Fig2 Liver disease-Forest map(random effect）


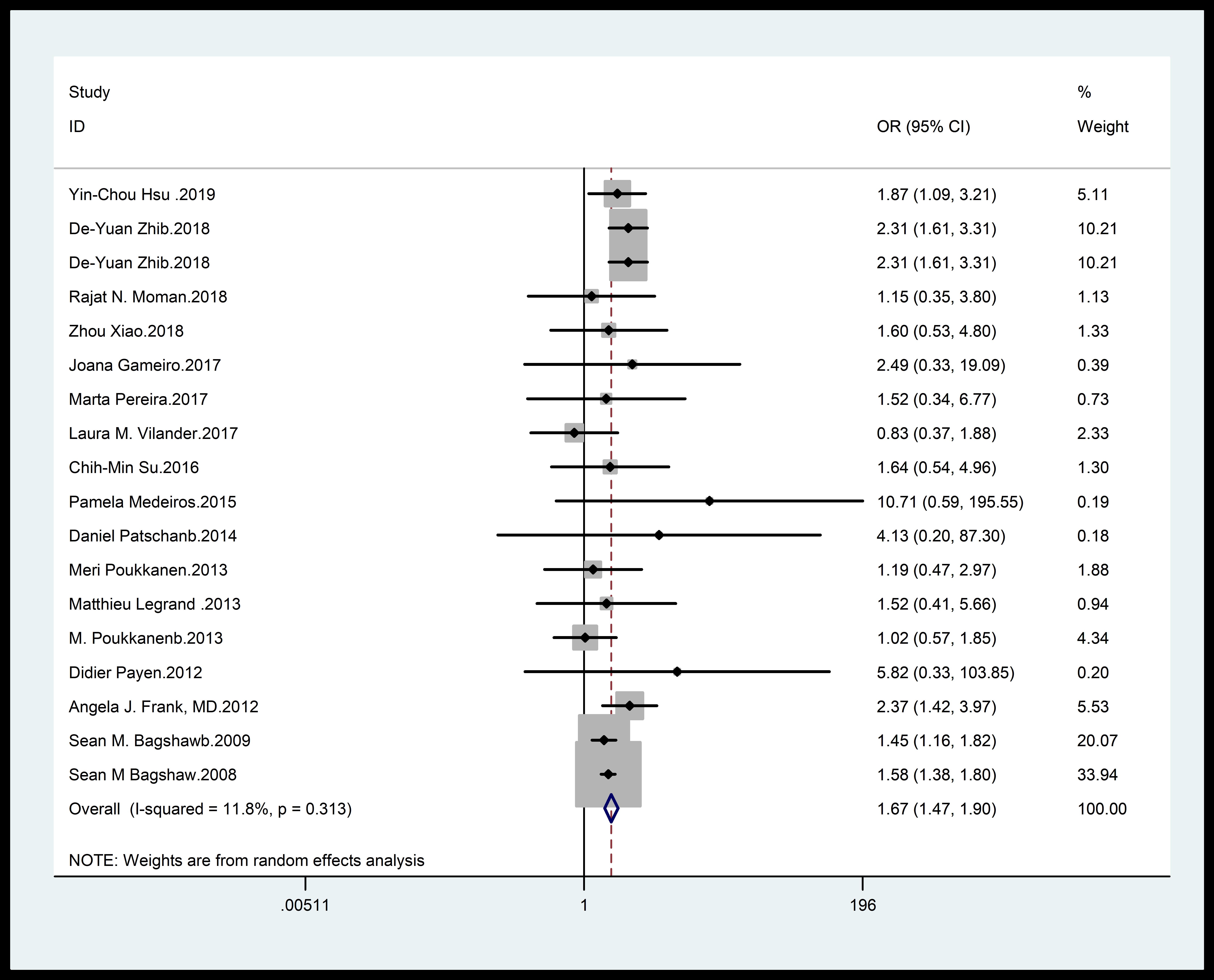


Fig3 Liver disease-Sensitivity analysis


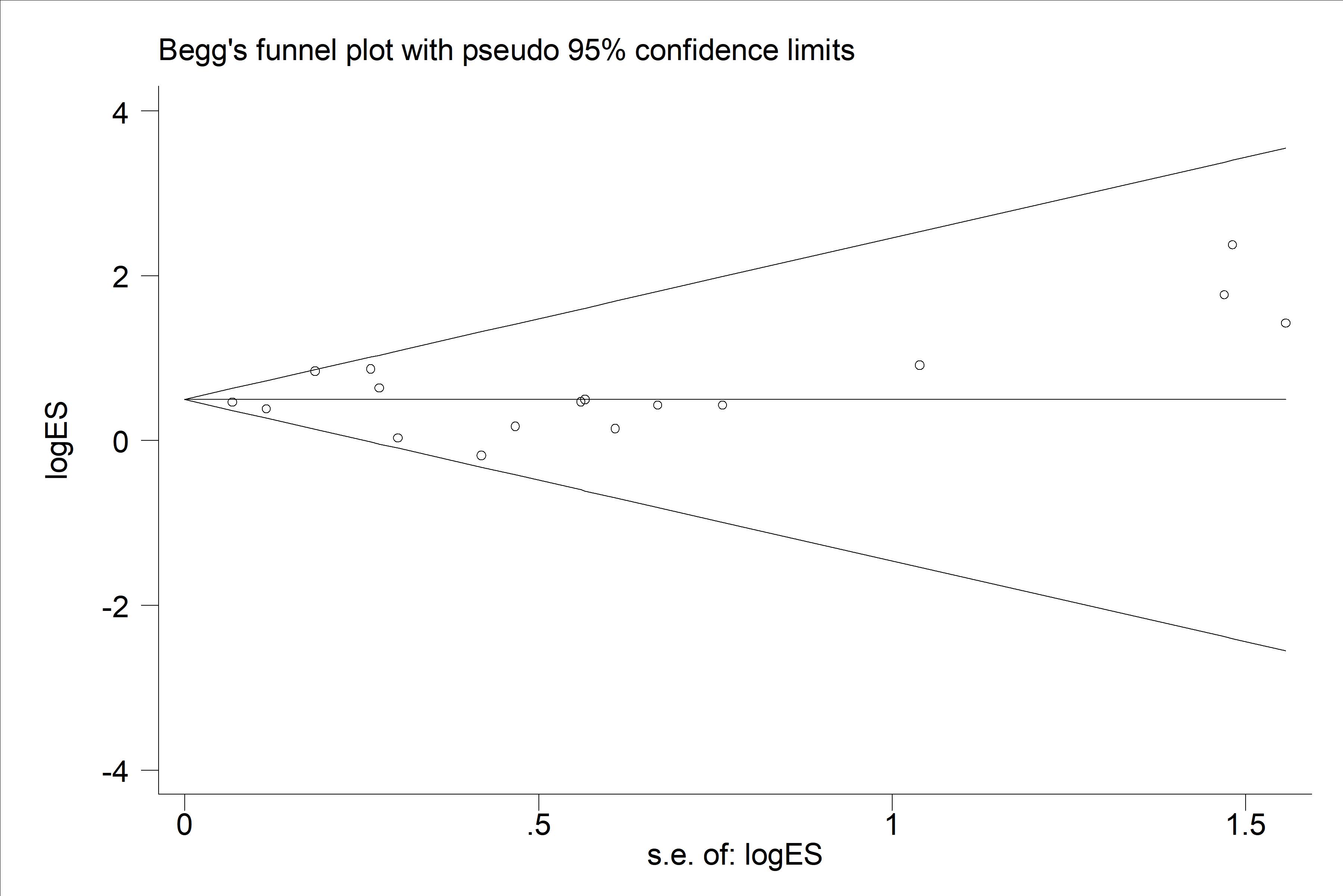

Supplement: Supplementary file 6 — Additional file 6. Fig. Liver disease-Forest plot and Sensitivity analysis. [file 12882_2020_1974_MOESM6_ESM.doc]
