# Supplementary material for: Rates, predictors, and mortality of sepsis-associated acute kidney injury: a systematic review and meta-analysis
Source: BMC Nephrol. 2020 Jul 31;21:318. doi: 10.1186/s12882-020-01974-8 (PMC7393862; doi:10.1186/s12882-020-01974-8)

Fig1 Abdominal infection-Forest map(Fixed effect)


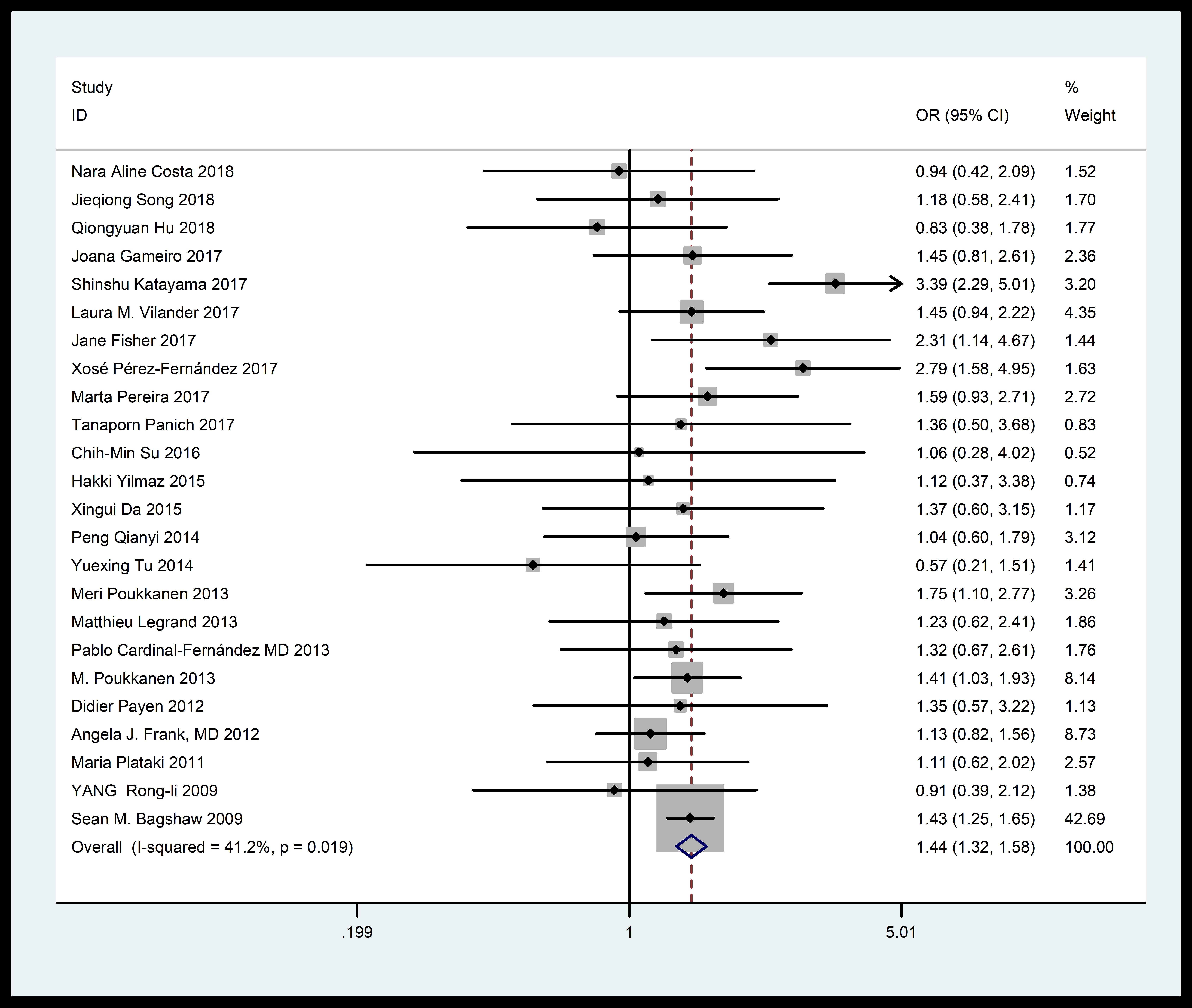


Fig2 Abdominal infection-Forest map(random effect)


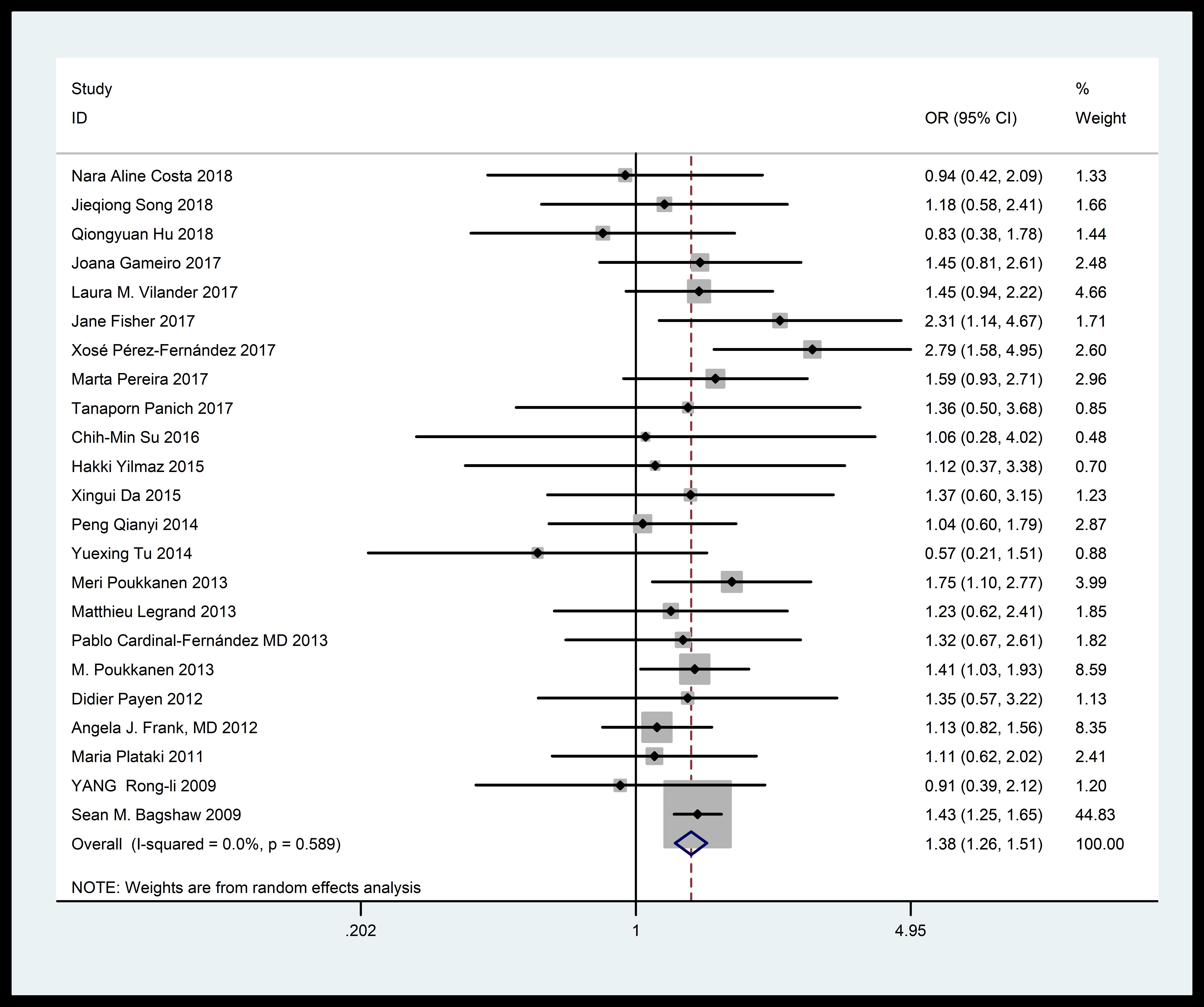


Fig3 Abdominal infection-Funnel plot


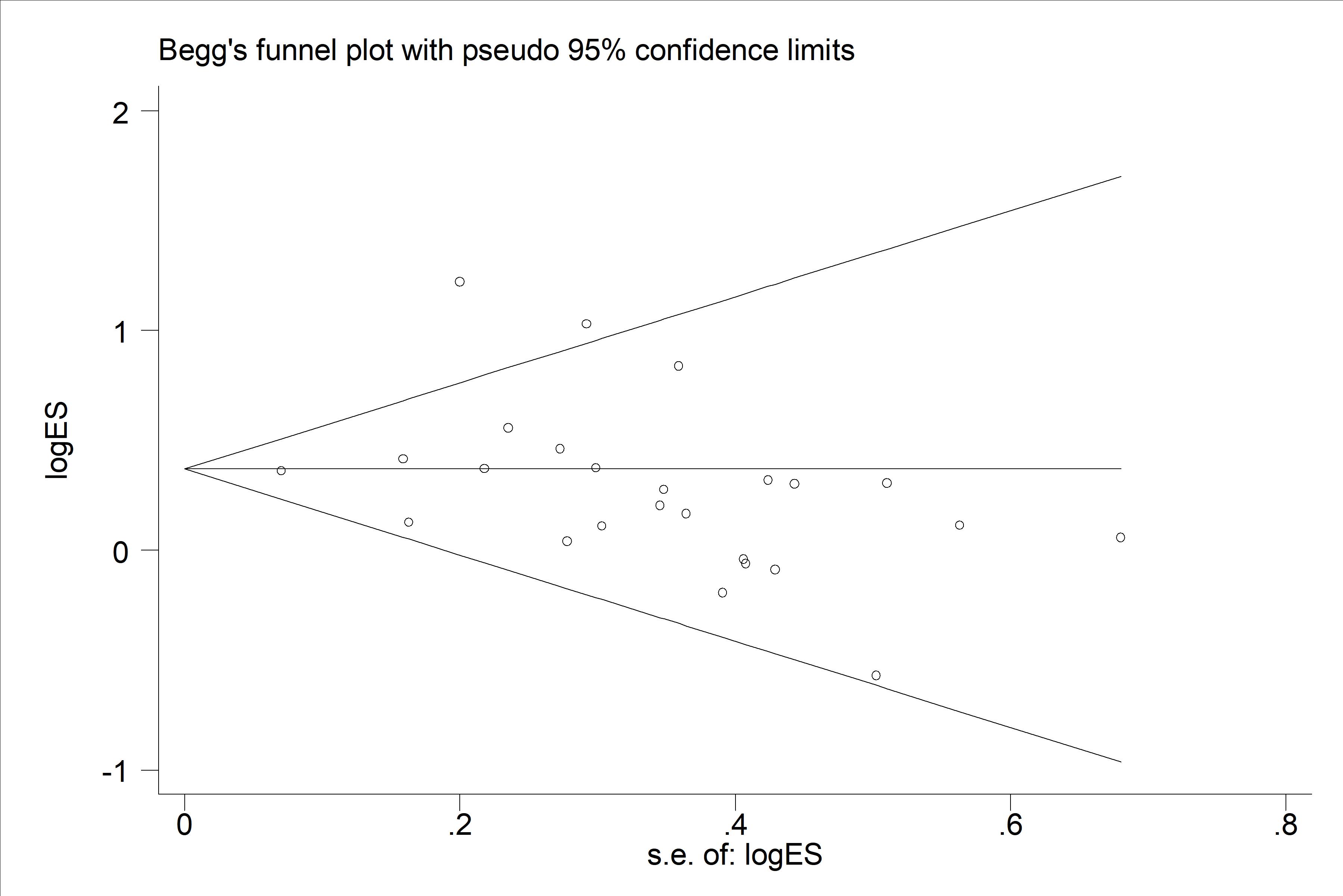


Fig4 Abdominal infection-Sensitivity analysis


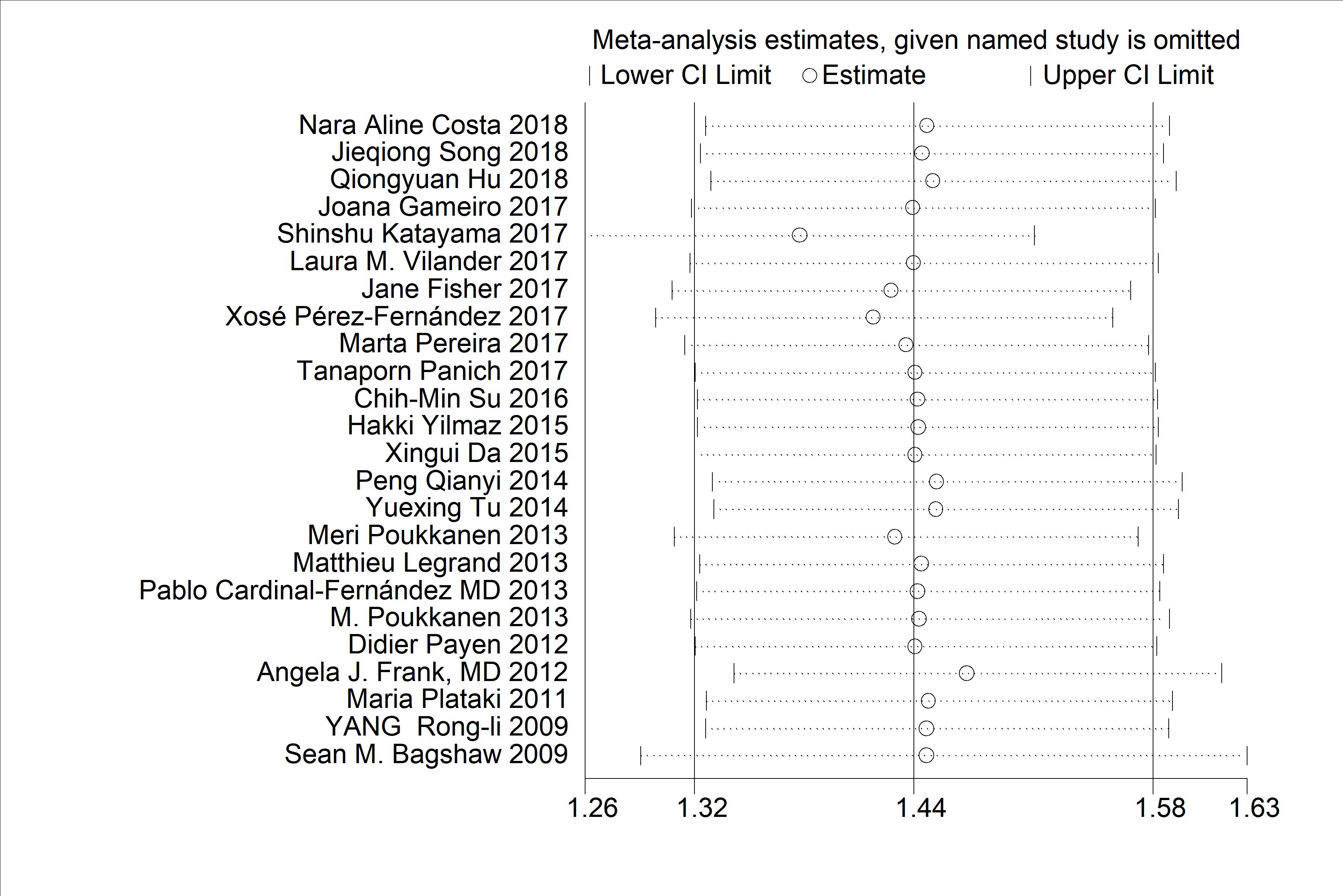

Supplement: Supplementary file 9 — Additional file 9. Fig. Abdominal infection-Forest plot, Funnel plot and Sensitivity analysis. [file 12882_2020_1974_MOESM9_ESM.doc]
