# Supplementary material for: Rates, predictors, and mortality of sepsis-associated acute kidney injury: a systematic review and meta-analysis
Source: BMC Nephrol. 2020 Jul 31;21:318. doi: 10.1186/s12882-020-01974-8 (PMC7393862; doi:10.1186/s12882-020-01974-8)

Fig1 Unknown source of infection-Forest map(Fixed effect)


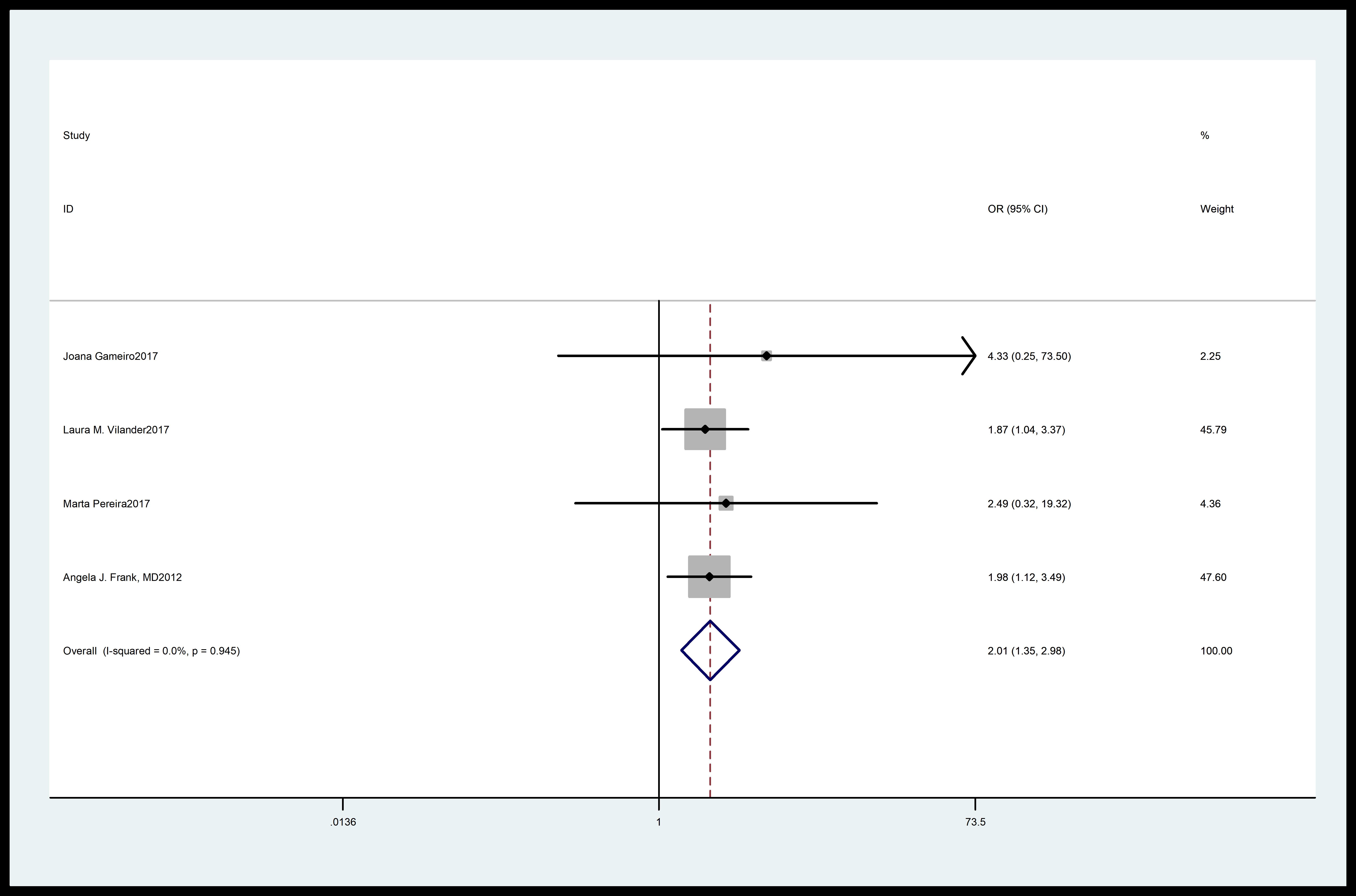


Fig2 Unknown source of infection-Forest map(random effect)


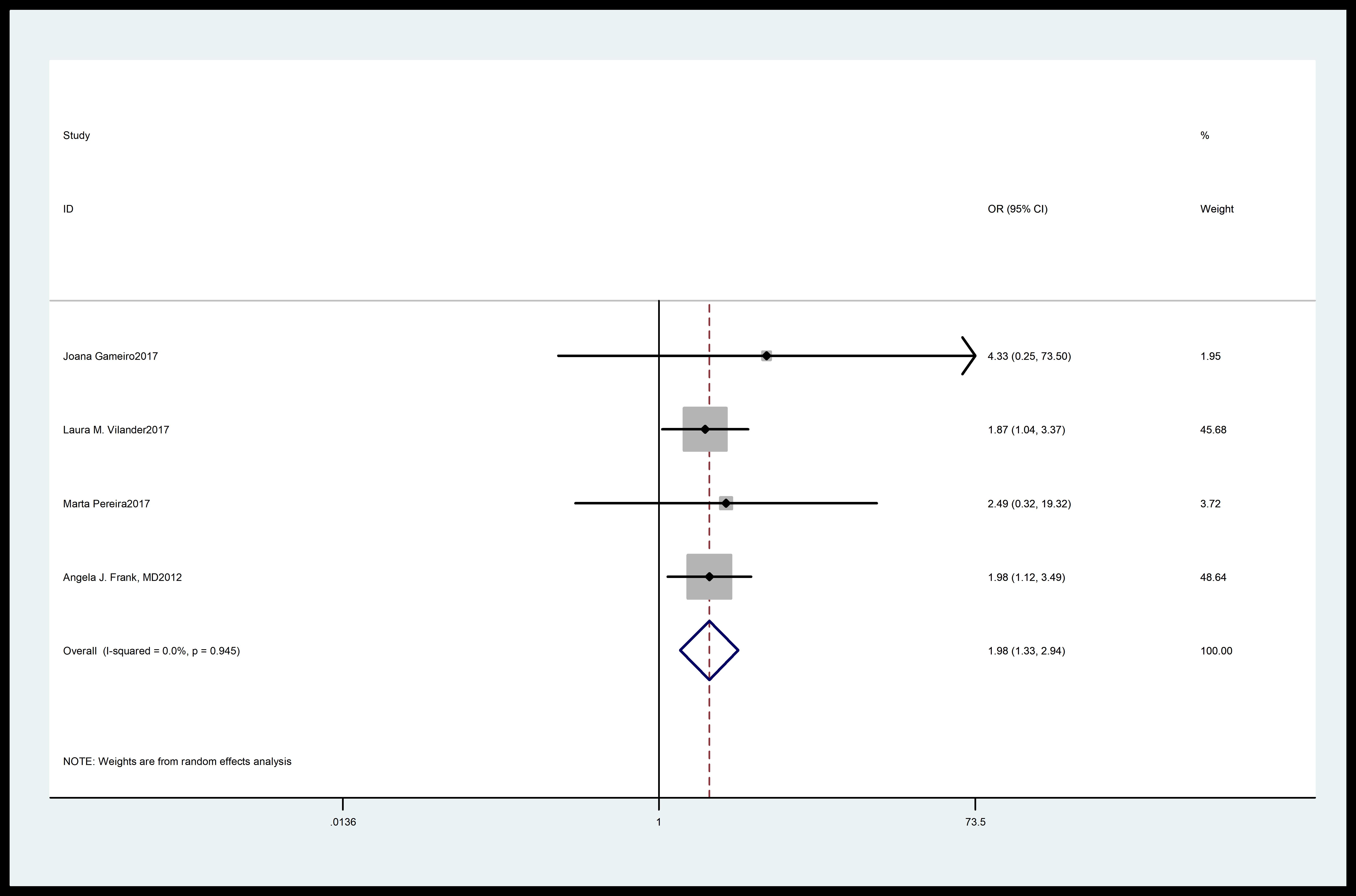

Supplement: Supplementary file 10 — Additional file 10. Fig. Unknown source of infection-Forest plot. [file 12882_2020_1974_MOESM10_ESM.doc]
