# Supplementary material for: Rates, predictors, and mortality of sepsis-associated acute kidney injury: a systematic review and meta-analysis
Source: BMC Nephrol. 2020 Jul 31;21:318. doi: 10.1186/s12882-020-01974-8 (PMC7393862; doi:10.1186/s12882-020-01974-8)

Fig1 Positive blood culture-Forest plot


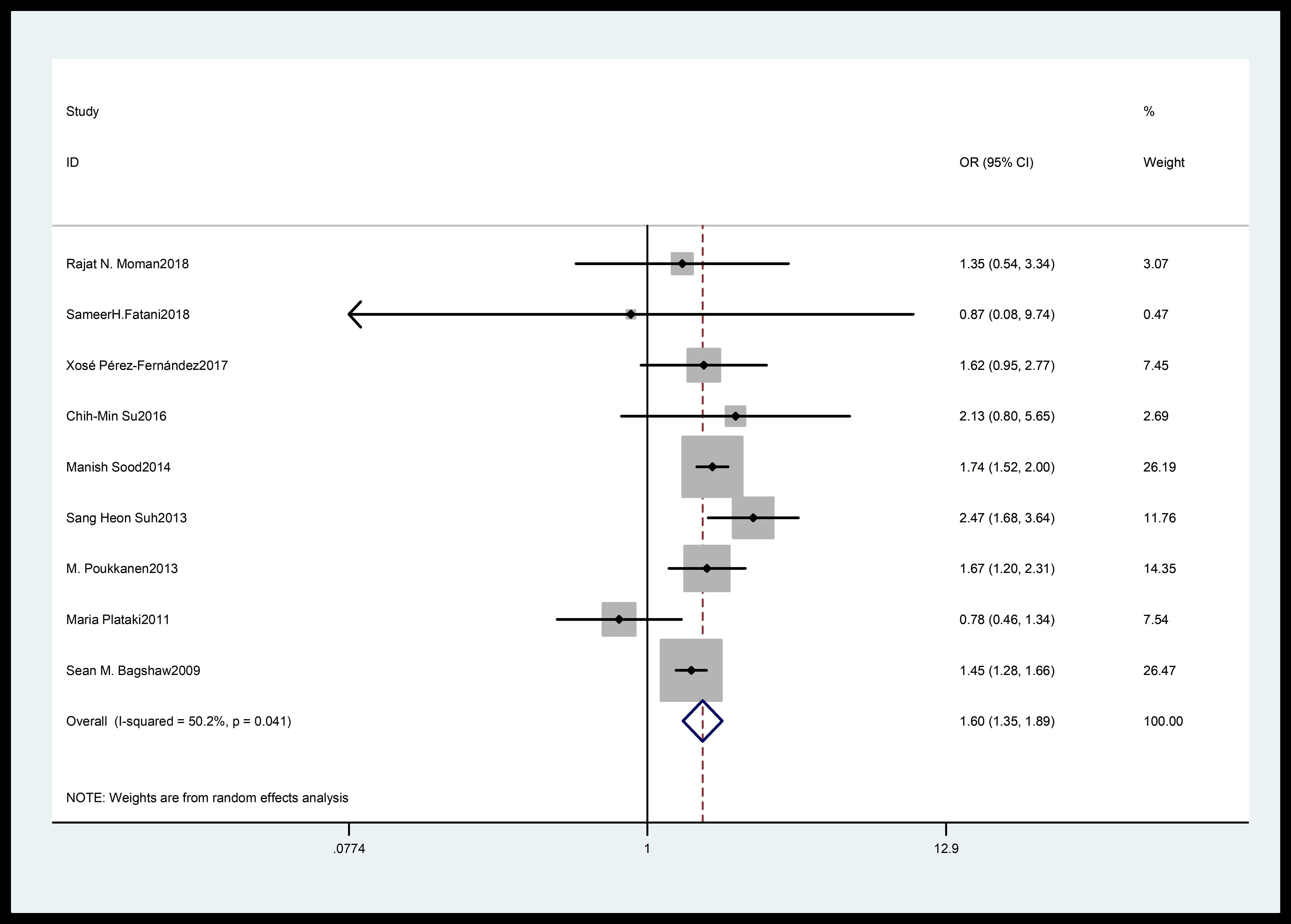


Fig2 Positive blood culture-Funnel plot


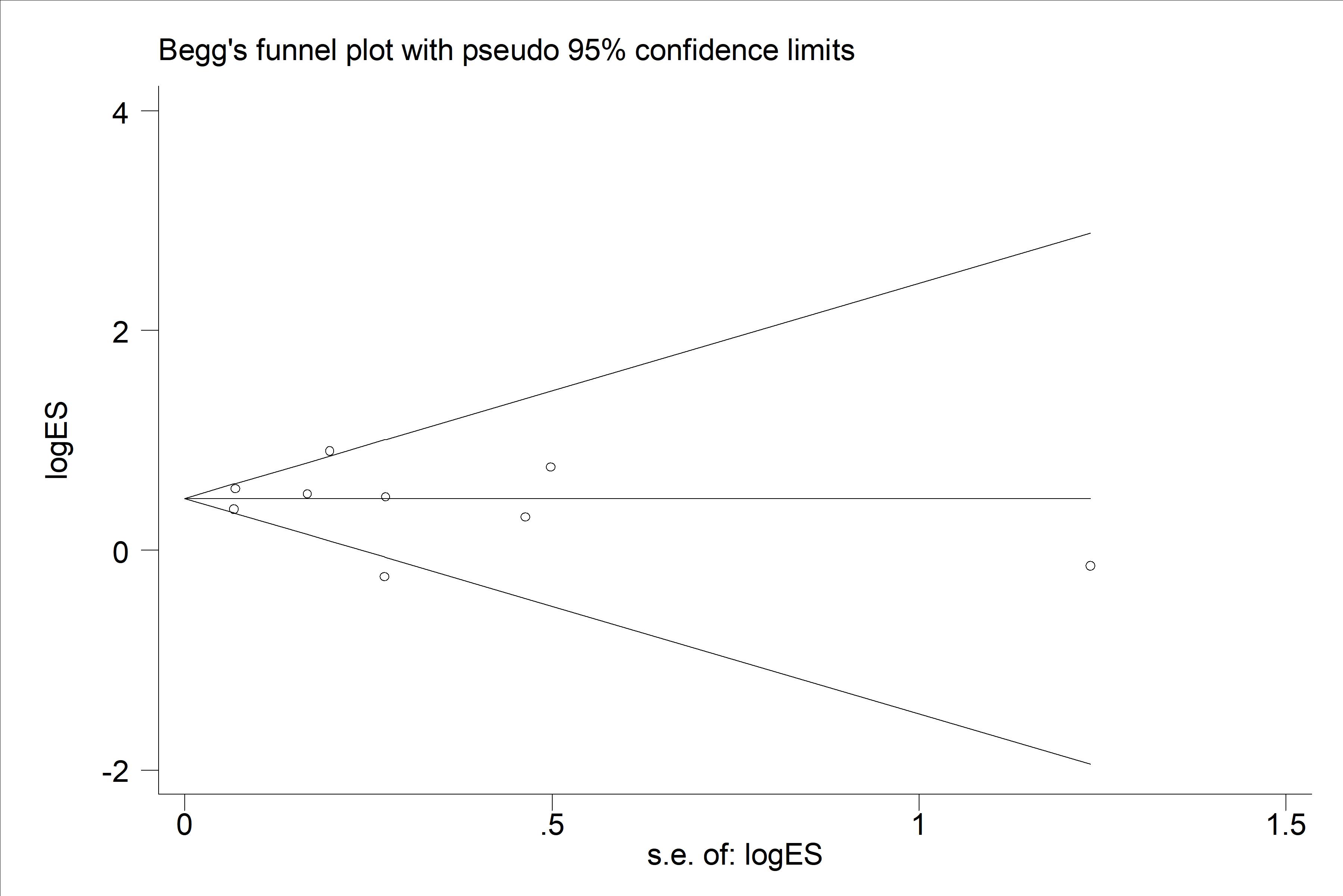
 Fig3 Positive blood culture-Sensitivity analysis


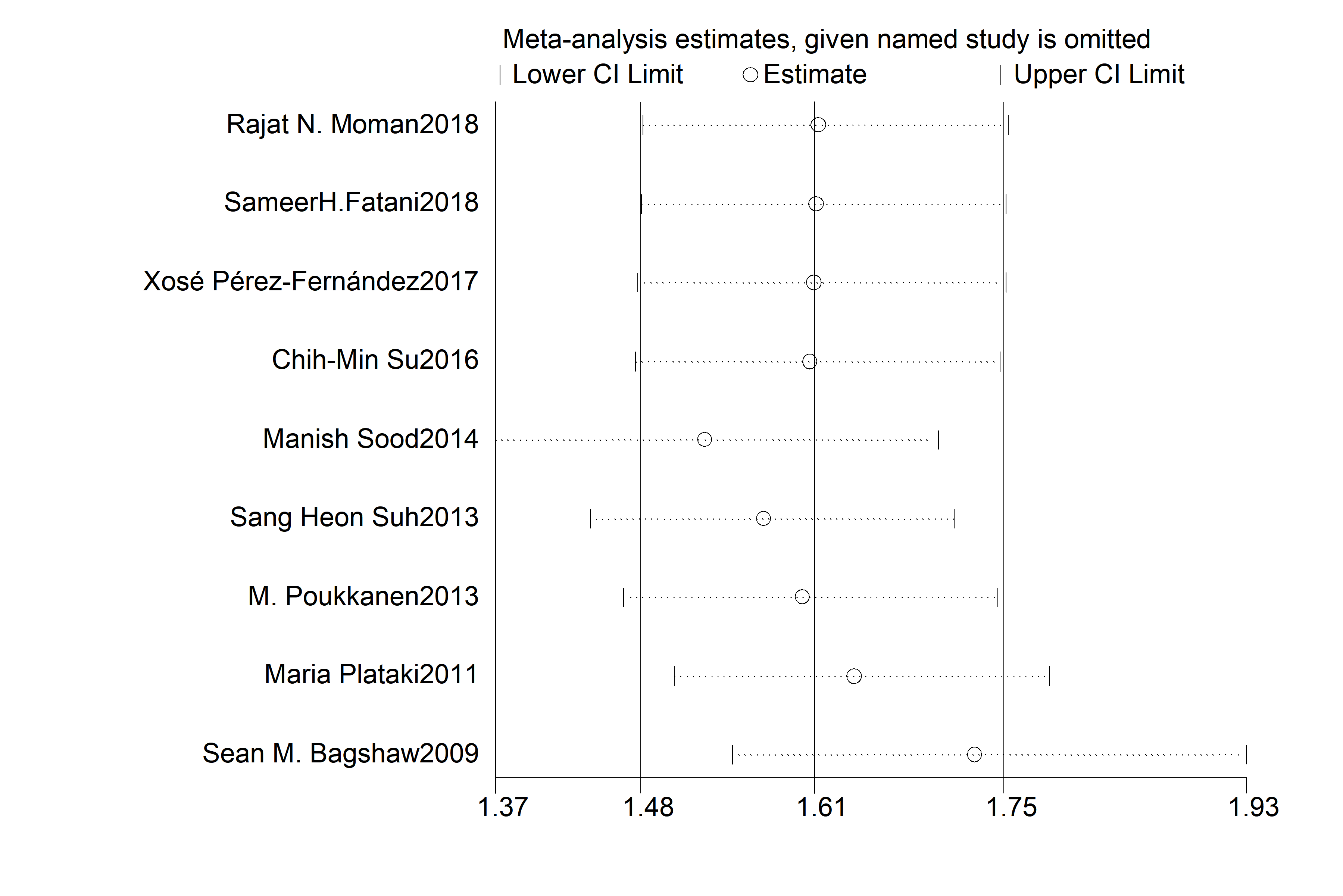

Supplement: Supplementary file 14 — Additional file 14. Fig. Positive blood culture-Forest plot, Funnel plot and Sensitivity analysis. [file 12882_2020_1974_MOESM14_ESM.doc]
