# Supplementary material for: Rates, predictors, and mortality of sepsis-associated acute kidney injury: a systematic review and meta-analysis
Source: BMC Nephrol. 2020 Jul 31;21:318. doi: 10.1186/s12882-020-01974-8 (PMC7393862; doi:10.1186/s12882-020-01974-8)

Fig1 Mechanical ventilation-forest map


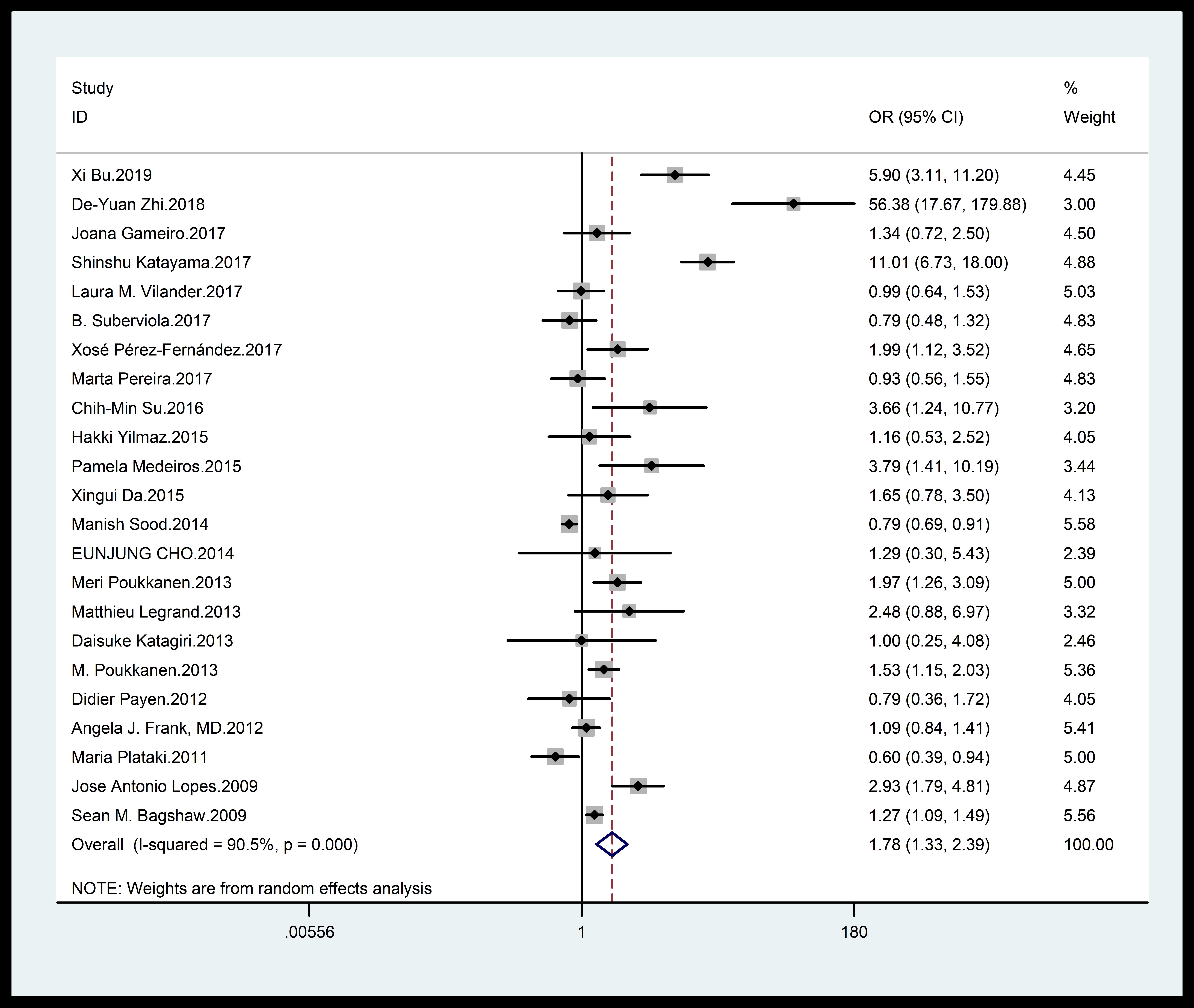


Fig2 Mechanical ventilation-Funnel plot


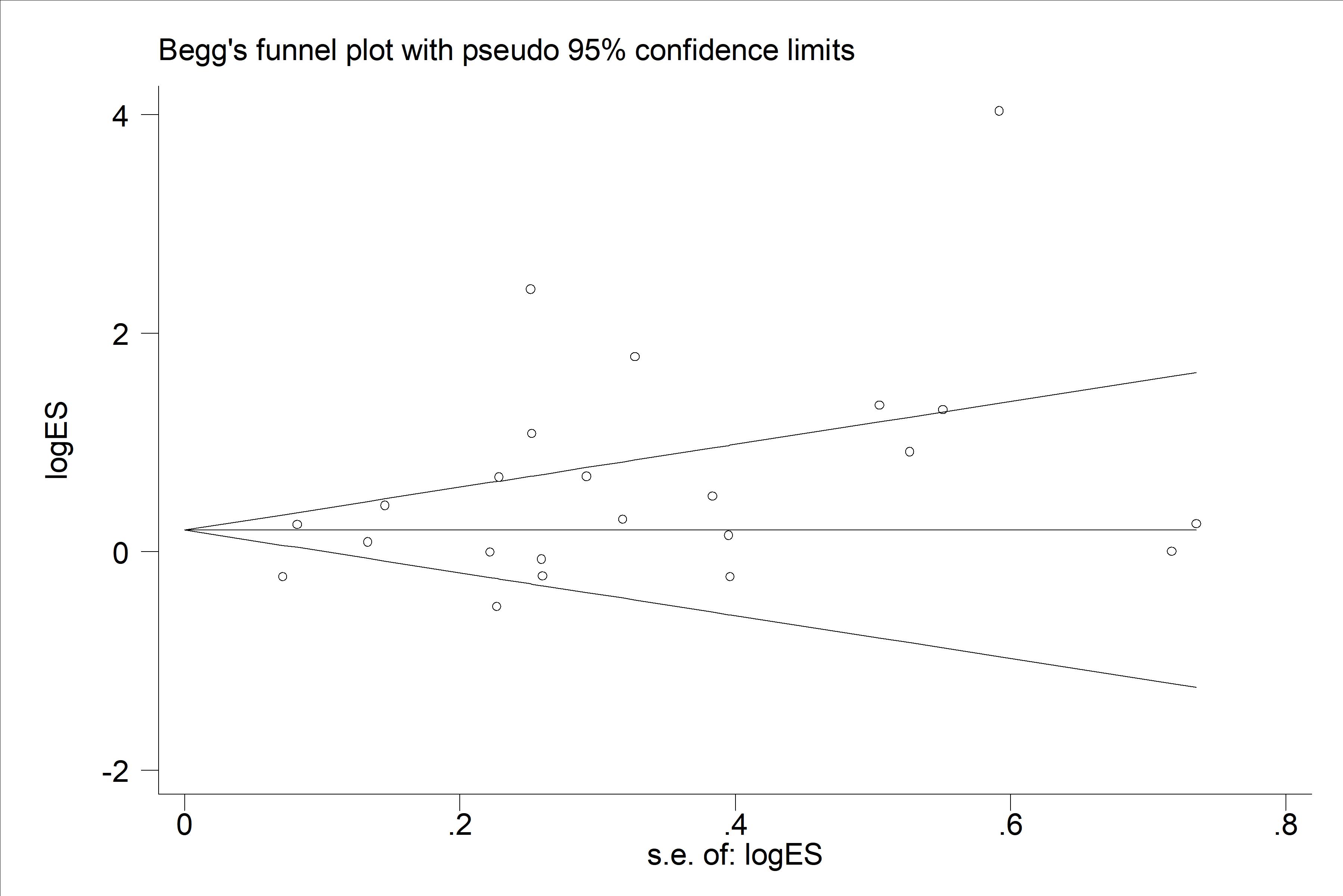


Fig3 Mechanical ventilation-Sensitivity analysis


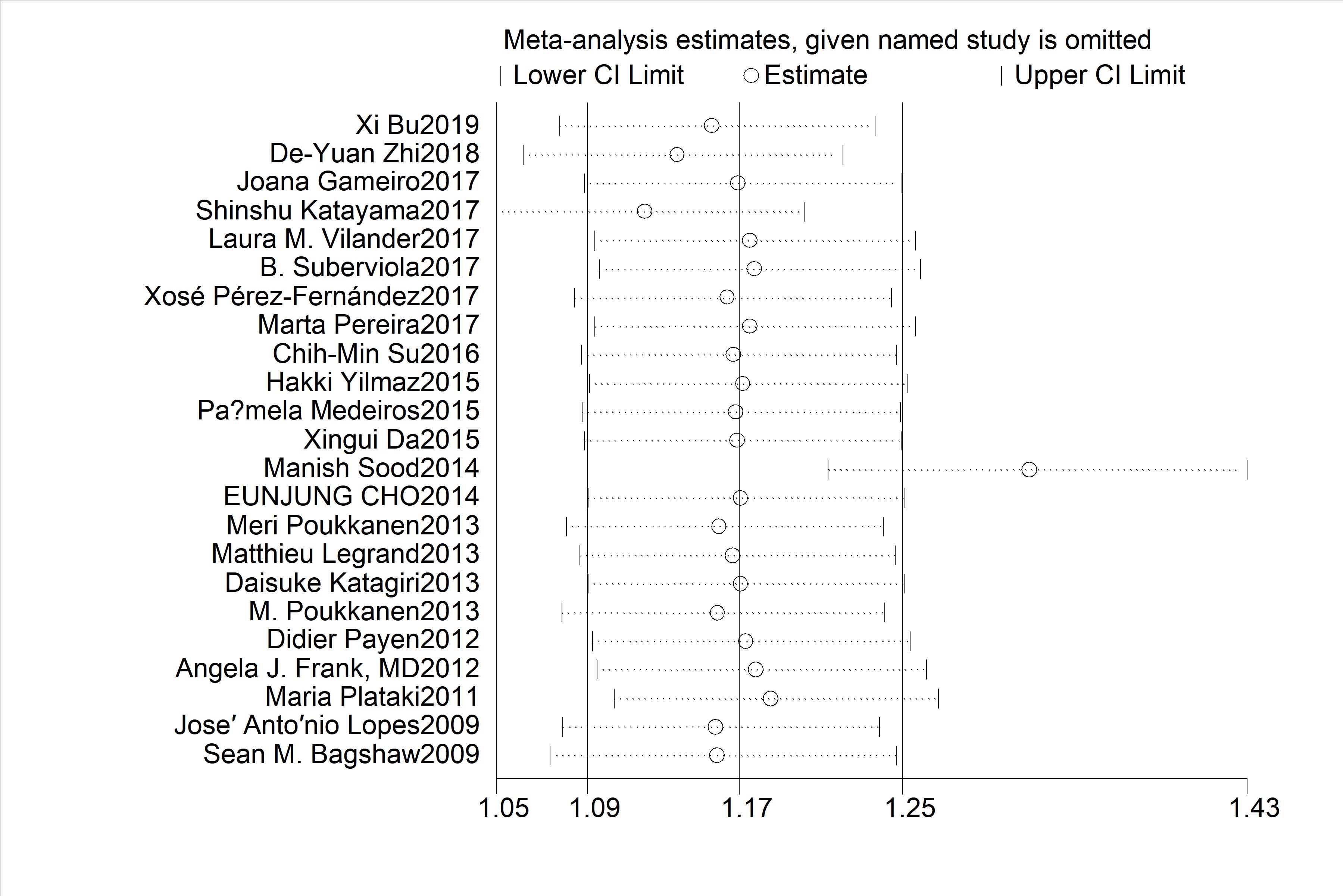


Fig4 Mechanical ventilation-Subgroup analysis


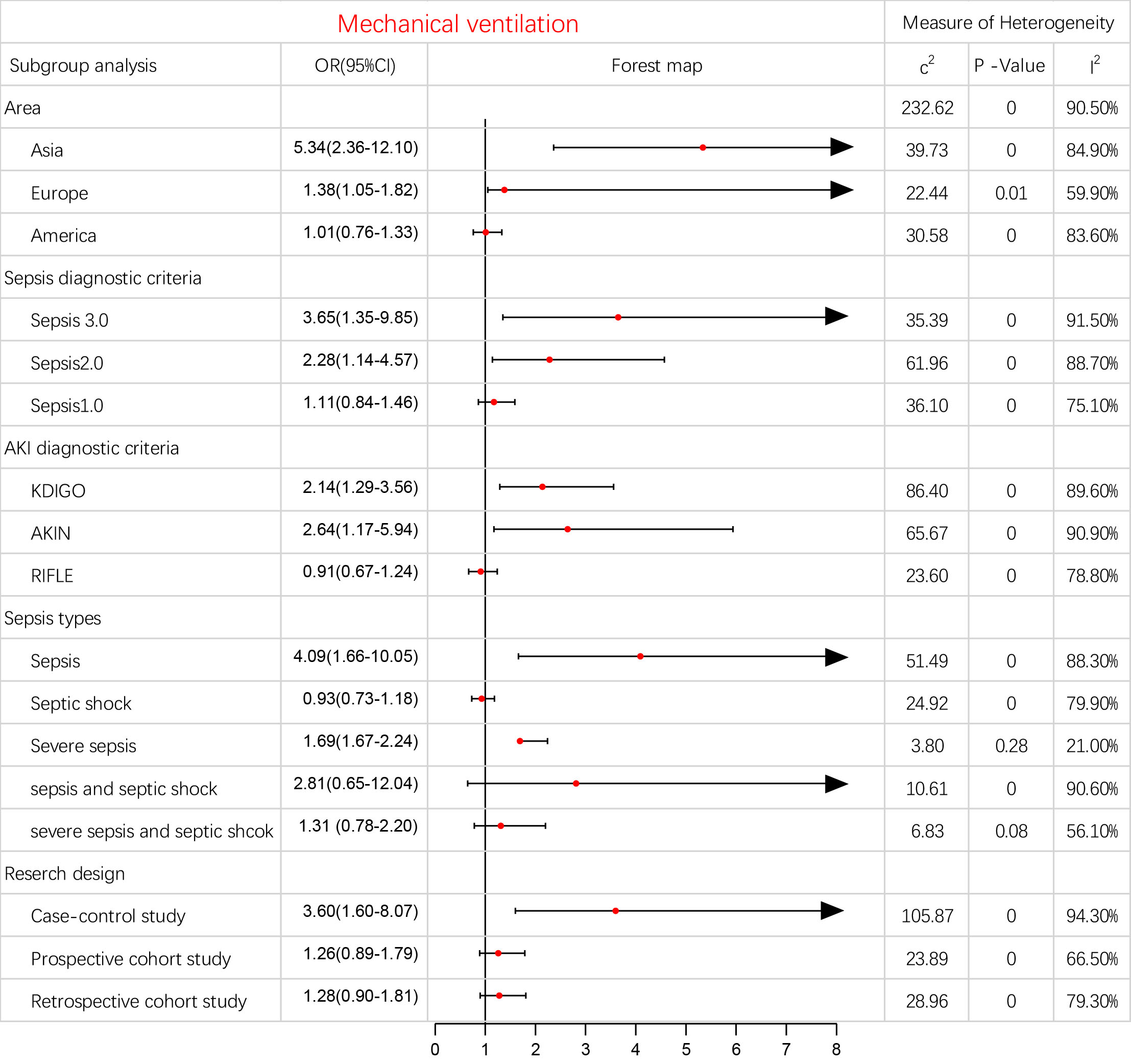

Supplement: Supplementary file 19 — Additional file 19. Fig. Mechanical ventilation-forest plot, Funnel plot, Sensitivity and Subgroup analysis. [file 12882_2020_1974_MOESM19_ESM.doc]
